# Neuropilin-1: A Key Protein to Consider in the Progression of Pediatric Brain Tumors

**DOI:** 10.3389/fonc.2021.665634

**Published:** 2021-07-01

**Authors:** Manon Douyère, Pascal Chastagner, Cédric Boura

**Affiliations:** ^1^ Université de Lorraine, CNRS, CRAN, Nancy, France; ^2^ Service d’Onco-Hématologie Pédiatrique, CHRU-Nancy, Nancy, France

**Keywords:** neuropilins, pediatric brain tumor, cancer stem cells, immune system, glioma, medulloblastoma

## Abstract

Neuropilins are transmembrane glycoproteins that play important roles in cardiovascular and neuronal development, as well as in immunological system regulations. NRP1 functions as a co-receptor, binding numerous ligands, such as SEMA 3 or VEGF and, by doing so, reinforcing their signaling pathways and can also interface with the cytoplasmic protein synectin. NRP1 is expressed in many cancers, such as brain cancers, and is associated with poor prognosis. The challenge today for patients with pediatric brain tumors is to improve their survival rate while minimizing the toxicity of current treatments. The aim of this review is to highlight the involvement of NRP1 in pediatric brain cancers, focusing essentially on the roles of NRP1 in cancer stem cells and in the regulation of the immune system. For this purpose, recent literature and tumor databases were analyzed to show correlations between NRP1 and CD15 (a stem cancer cells marker), and between NRP1 and PDL1, for various pediatric brain tumors, such as high- and low-grade gliomas, medulloblastomas, and ependymomas. Finally, this review suggests a relevant role for NRP1 in pediatric brain tumors progression and identifies it as a potential diagnostic or therapeutic target to improve survival and life quality of these young patients.

## Introduction

Neuropilins (NRPs) are transmembrane glycoproteins found in all vertebrates and are highly conserved across species. Two homologous NRPs isoforms have been identified, namely neuropilin-1 (NRP1) and neuropilin-2 (NRP2). NRPs are non-tyrosine kinase surface proteins that play important roles in neuronal development, cardiovascular development, and in the immune system (1–3). NRPs are co-receptors involved in a wide variety of signaling pathways and have pleiotropic effects on axon guidance, immune responses, angiogenesis, cell survival, migration, and invasion (1–7). NRPs were originally discovered as surface proteins involved in neuronal development by participating to semaphorins axonal guidance (8, 9). They were later identified as a key protein in vascular development because of their interaction with vascular endothelial growth factor A (VEGFA or VEGF) (10). Studies over the past decades have revealed their involvement in a wide range of physiological activities; however, more recent studies have highlighted their role in pathological processes, such as cancers (6, 7, 11). NRPs are overexpressed in a wide variety of cancers, such as pediatric brain tumor, and their overexpression is correlated with a poor prognostic for those patients (12–16).

In children, primary tumors of the central nervous system (CNS) are the second most common cause of cancer after leukemia, accounting for approximately one fourth of all childhood cancers. There are approximately 5,000 new cases diagnosed each year in the United States among children younger than 18 years (17). Actually, this term encompasses a wide range of very different tumors, with different treatment and prognosis. Pediatric brain tumors are equally distributed between supra- and infra-tentorial regions. The most frequent tumors are represented by gliomas and embryonic tumors of the CNS. Although their outcomes have improved over the last few decades, thanks to the combined uses of surgery, radiotherapy, and chemotherapy, those tumors still represent the leading cause of disease-related death in children, and suboptimal long-term outcomes are still frequent, especially in young ages. Indeed, in comparison with age-matched patients who survived extra CNS malignancies, pediatric brain tumor survivors usually present severe cognitive, neurological, endocrine, social, and emotional impairments. It is a crucial need to find new diagnostic markers and new efficient therapeutic strategies by using recent tools to detect important genes and signaling pathways that serve to drive tumor proliferation and which could then be targeted by therapies.

Although the roles of NRP1 in tumor angiogenesis, tumor microenvironment, as well as its possible targeting in tumor progression, have recently been reviewed elsewhere (18–20), in this review, we summarize the up-to-date knowledge on NRP1 and its role in pediatric brain tumors progression. In particular, we detail the role of NRP1 in cancer stem cells and immune system cells during tumorigenesis.

## Pediatric Brain Tumors

### Gliomas

Gliomas are the most frequently pediatric brain tumors and represent 25% of all brain tumors. They are heterogeneous and are divided into two main categories, low- and high-grade tumors, according to their aggressiveness and their prognosis. Low-grade gliomas (LGGs), such as pilocytic astrocytoma (PA), are usually curable while high-grade gliomas (HGGs), such as diffuse intrinsic pontine glioma (DIPG), are consistently fatal.

LGGs, classified by the World Health Organization (WHO) as grade I or II, comprise several subgroups, including PA, pleomorphic xanthoastrocytomas (PXA), pilomyxoid astrocytomas (PMA), subependymal giant cell astrocytomas (SEGA), low-grade fibrillary astrocytomas, and diffuse astrocytomas. The most common LGG diagnosed in children aged 0 to 14 years is the PA, which accounts for 85% of all LGGs (21). Five-year overall survival is approximately 95% (22), with the exception of tumors with BRAF V600F mutants, which have a higher tumor progression potential and poorer outcome (23). The majority of LGGs exhibit alterations in the mitogen-activated protein kinase (MAPK) signaling pathway, which has led to the targeting of this pathway by MAPK inhibitors. In addition, BRAF mutations are commonly found in LGGs, which has led much research into the therapeutic benefits of BRAF inhibitors. In contrast to LGGs in adults, IDH mutations are almost absent in childhood tumors, and malignant progression is rare (24, 25). The current treatment strategy for LGGs is mainly based on the surgical excision of the tumor, which may prove curative in case of total resection. In the cases of a tumor progression during partial surgical resections, chemotherapy treatments, such as vincristine and carboplatin combination or vinblastine monotherapy, are used. Chemotherapy leads to a progression-free survival (PFS) of 30% to 50% (25). BRAF V600E mutations and CDKN2A deletions are associated with a worse prognosis, with a 10-year PFS of 27% (26). New clinical trials are using molecular profiling to stratify patients and test the efficacy of MEK inhibitors for tumors with BRAF fusions, and of MEK, BRAF, or combined inhibitors for tumors with BRAF V600E mutations. The efficacy of mTOR inhibitors has now been clearly demonstrated in the treatment of SEGA (27). HGGs represent 8-12% of all childhood brain tumors. They are most often represented by diffuse midline gliomas and DIPGs, followed by hemispheric HGGs. Their prognosis remains bleak, with a long-term survival rate of less than 10%. Outcome does not appear to be correlated with the WHO grade of the pediatric tumors. HGGs are phenotypically similar to adult diseases, but molecular studies suggest that the molecular signatures are different (28, 29). The new generation of genomic sequencing has uncovered characteristic alterations in HGGs, such as mutations at position 27 (K27M) in the genes coding for histone 3, or G34R/V mutations (30, 31). The molecular alterations coding for the histone variants H3.3 and H3.1 are present in about 80% of midline gliomas. Other molecular characteristics in HGGs are TP53 mutations (> 85%) and MGMT promoter methylation (32). NTRK gene fusions have been analyzed, which could be targeted by selective TRK kinase inhibitors (33). Standard treatment of HGGs consists of total surgical resections when possible, associated with radiotherapy and chemotherapy. However, to date, the role of chemotherapy remains undefined (34). Patients with H3.1 mutant tumors have a better prognosis than those with H3.3 mutant tumors (35). K27M mutant diffuse midline gliomas and DIPGs are still fatal because surgical resections, even partial, are mostly impossible, because radiation therapy offers only temporary improvement, and because no chemotherapy or targeted therapy has demonstrated a survival benefit.

### Medulloblastomas

Medulloblastoma (MB) is the most common malignant brain tumor of the posterior fossa in pediatric, accounting for 15% to 20% of all childhood brain tumors. The survival rate of patients with embryonal tumors of the central nervous system, such as MB, is 54% at 5 years. The prognosis is significantly worse before the age of 1 year: 62% survival rate at 5 years, compared to 81% for children aged 10 to 14 years (36, 37). Because of its embryonic origin, MB occurs more frequently in children than adults, and accounts for only 1% of all adult brain tumors. The 5-year overall survival rate of MB patients is estimated at 75% to 80% in the absence of metastases. Unfortunately, despite treatment, recurrences remain frequent, especially when metastases are present at the time of diagnosis (30% of cases). MB develops within the cerebellum, and some of these tumors will metastasize and disseminate mainly to the leptomeningeal surface of the brain and to the spinal cord. The WHO has defined a histopathological classification for MBs, distinguishing between four groups: classic MBs, desmoplastic/nodular MBs, MBs with extensive nodularity, and large cell/anaplastic MBs. However, this classification is not sufficient to determine the prognosis and the choice of treatment. The WHO studies have highlighted four subgroups of MBs: the WNT subgroup (approximately 10% of MBs), and the sonic hedgehog (SHH) subgroup (approximately 30%), associated with a better prognosis, in contrast to subgroups 3 (approximately 25%) frequently associated with metastasis, and 4 (about 35%) the most aggressive MBs, characterized by a high recurrence rate due to a high potential for metastatic dissemination (38). Current treatments are based on prognostic factors which have led to define four risk groups: low-risk group, associated with a survival rate above 90% (mainly MB of the WNT subgroup); standard-risk group, associated with a survival rate between 75% and 90% (SHH subgroup of MB and group 4 metastatic MB); high-risk group (survival rate between 50% and 75%) and very high-risk group (MYC-amplified group 3 patients with metastatic disease or SHH tumors with TP53 mutations), both associated with a survival rate of less than 50% (in particular group 3 and SHH MB with the TP53 mutation) (39). Treatment begins with surgical resection as complete as safely possible, followed by radiotherapy with or without (for children older than 5 years in the standard-risk group) chemotherapy. The conventional dose of the craniospinal radiotherapy is 36 Gy for high-risk group patients, and 24 Gy for standard-risk group patients, followed by a 54 Gy boost to tumor bed. For low-risk group patients, current clinical trials are designed to de-intensifying the dose of craniospinal radiotherapy in an attempt to reduce long-term side-effects. In patients younger than 3 to 5 years, radiotherapy tends to be avoided and replaced by an intensive chemotherapy (40). Significant side-effects, secondary to surgical resection, radiation therapy, and chemotherapy, are frequently observed and include cerebellar mutism, neurocognitive deficits, hearing loss, and endocrine pathologies (41).

### Ependymomas

Ependymomas (EPNs) are the third cause of malignant brain tumors in children. These tumors affect mainly the children younger than 5 years. EPNs are usually located in the posterior fossa (70% of cases), but they can develop anywhere within the central nervous system. The histological grade score of the tumor is not reproducible and has no prognostic impact. Recent advances in molecular biology confirm that, depending on their localization (spinal, supratentorial, or posterior fossa), EPNs are different diseases. These tumors have a worse prognosis in children than in adults (38, 42). EPNs are characterized by a fusion between the C11ORF95 and RELA genes in 72% of cases. This alteration leads to the sequestration of the fusion protein within the nucleus and results an aberrant activity of the oncogenic NF-κB signaling pathway (43). Pediatric supratentorial EPNs (ST-EPNs) are dominated by the subgroups ST-EPN-RELA and ST-EPN-YAP1, while the subgroups PF-EPN-A and PF-EPN-B are predominant in posterior fossa ENPs (PF-EPN). In supra tentorial locations, YAP1-fused EPNs are correlated with a better prognosis (44). Conversely, a poor prognosis is frequently associated with PF-EPN-A (survival rate < 50%). PF-EPN-B occurs in older children and adults and has an excellent outcome (45). The current treatment strategy for EPNs is a surgical tumor resection, whose extensions is a major prognostic factor, always followed by radiotherapy (59 Gy to the tumor bed), except for children younger than 1 year. As with gliomas, the efficiency of chemotherapy is still debated and is the object of current clinical trials.

### Other Pediatric Brain Tumors

There is a wide diversity of pediatric brain tumors that are very rare, accounting for less than 30% of all tumors. Some of them, such as the choroid plexus papillomas, a benign (WHO grade I) neuroepithelial intraventricular tumor, have an excellent prognosis after complete surgical resection when they occur in children. Other tumors, such as craniopharyngiomas, arising from the sellar region, also have a very good overall survival after complete resection, but relapses may later occur that necessitate a complementary treatment by radiotherapy. The excellent overall survival of these tumors is unfortunately impaired by endocrine, and often visual, sequelae.

Other embryonal tumors of the CNS, like atypical teratoid and rhabdoid tumors, and embryonal tumors with multilayered rosettes, are highly aggressive, poorly differentiated, and occur predominately in young children. Their treatment is therefore very challenging, and their prognosis is still grim.

Unfortunately, despite the progress made in recent decades in the management of these cancer types, the effectiveness is still suboptimal and sequelae are frequent. Indeed, chemotherapy has been shown to provide significant toxicities and adverse side effects (46, 47). Similarly, radiotherapy induced many late side effects, especially for young patients, such as neurocognitive and neuroendocrine deficits, bone and soft tissue hypoplasia, and secondary benign or malignant tumors (47, 48). The current challenge is to improve the survival rate of patients with pediatric brain tumors while minimizing the toxicity of these treatments. There is, therefore, an urgent need to improve the treatments for brain tumors by finding new potential therapeutic targets. High resolution genomic, epigenetic, and transcriptomic profiling define various sub-classifications, according to tumor location, patient age, and prognosis, and suggest the possibility to develop more adapted treatments.

## Neuropilin-1

### Physiological Functions and Structure

**Figure 1:**
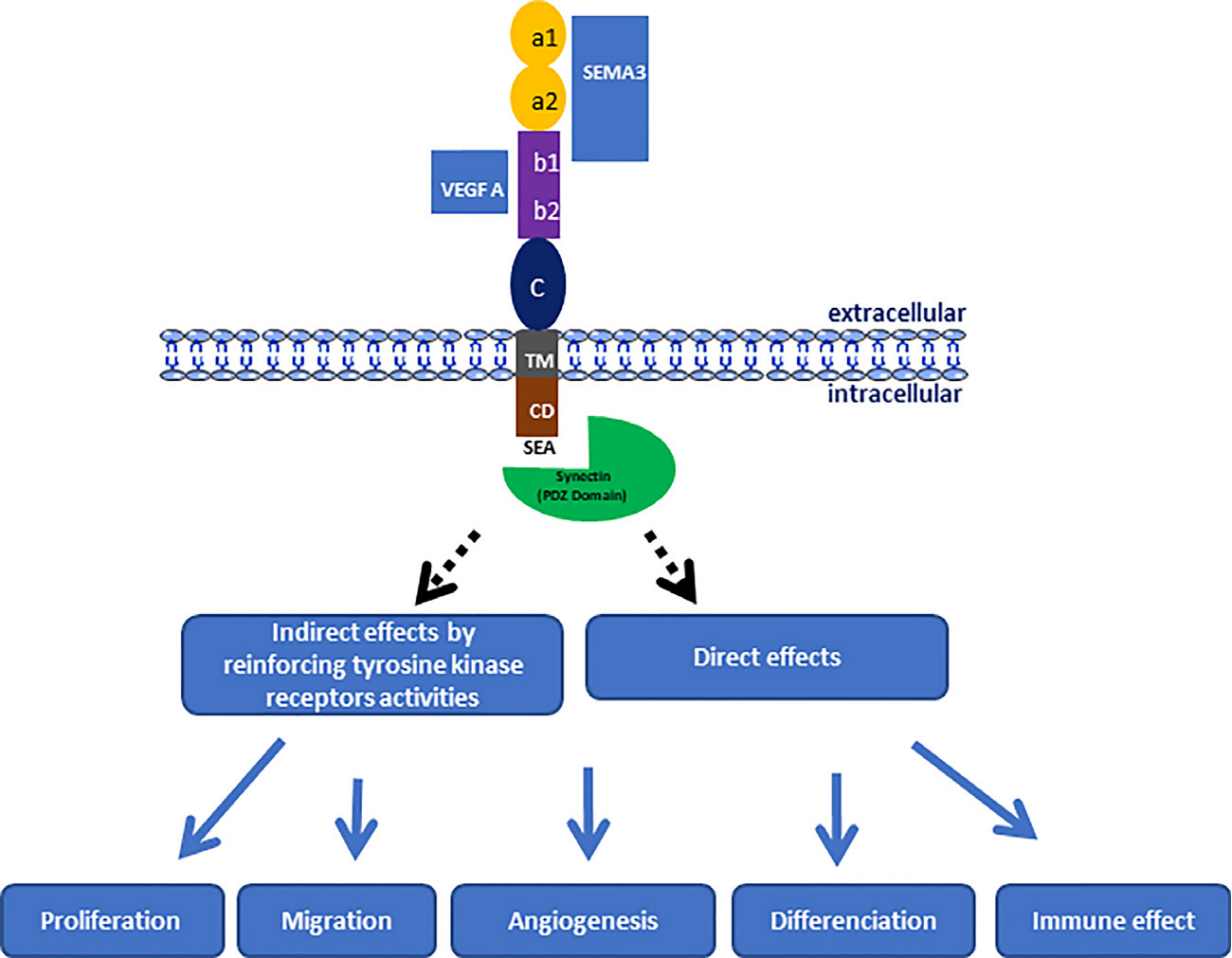
Schematic representation of NRP-1 structure indicating the link regions of natural ligands and its involving in the progression of pediatric brain tumors through the control of different biological process.

NRPs are transmembrane glycoproteins specific to vertebrates. There are two members in this family of proteins, NRP1 and NRP2, which are coded by two different genes located on two chromosomes (10p12 for NRP1 and 2q34 for NRP2). NRPs play an important role in biological processes, such as neuronal development, cardiovascular development, and immunological system (1–3). Overexpression of NRP1 causes fetal death in utero of chimeric mice, and the embryos show significant dysfunctions in neuronal development, with ectopic sprouts and defasciculation of nerve fibers (1). Likewise, NRP1 has been shown to be involved in cardiovascular and vascular development, because its abnormal expression in mouse embryos causes anomalies in the cardiovascular system and a dysfunction in blood vessels and capillary formation (1, 2). NRPs are expressed in various cell types, such as neuronal cells, endothelial cells, immune cells, smooth muscle cells, and also tumor cells (1, 3, 10). NRP1 is a multifunctional co-receptor capable of binding to different ligand families involved in diverse biological pathways (**Figure 1**). Initially, NRPs were mainly known for their key role in axonal guidance mediated by SEMA proteins with their receptors, plexins (2, 49). Subsequently, NRPs were identified as co-receptors of the VEGF family and their receptors (VEGFRs), enhancing the signaling pathway and thus promoting angiogenesis (10, 50).

NRP1 measures 130 to 140 kDa, sharing 44% sequence homology with NRP2, and both glycoproteins have the same structural organization in three protein domains (**Figure 1**). NRPs are composed of N-terminal extracellular domains, a transmembrane domain and an intracellular domain. The extracellular domains are composed of three subdomains, namely a1a2 (CUB), b1b2, and c. The extracellular domains a and b are both involved in ligand binding, whereas domain c mediates the homo- and hetero-dimerization of NRPs, which seems essential for their functions. Moreover, it has been shown that the a1a2 domain of NRP1 corresponds to the binding domain of SEMA, while the b1b2 domain is able to bind ligands of the VEGF family (51) (**Figure 1**). NRP1 has no kinase activity of its own but enhances the kinase activity of co-receptors, such as VEGFRs or plexins. Moreover the intracellular domain ends with a consensus sequence (SEA) that allows it to interact with the PDZ protein domains of cytoplasmic proteins, such as synectin (52, 53). Indeed, synectin, also named GIPC1 (GAIP-interacting protein C terminus member 1), is required for stable receptors complex formation (54), and could act directly on different signaling pathways *via* the RhoA GTPase (55), as well as on cytoskeletal networks for the internalization of α5β1 integrins (56).

### NRP1 Ligands and Co-Receptors

#### Semaphorins and Plexins

NRP1 was originally identified for its role in the nervous system development, particularly through its function as a receptor for SEMA, especially SEMA 3 (49). SEMA are grouped into eight numerically named classes, and only classes 3, 4, 6, and 7 are present in vertebrates. SEMA 3 is one of eight subgroups of the protein family that have a role in guiding axon growth as soluble chemorepellents, which is essential for the structuring of the nervous system (49, 51). They are also involved in cell apoptosis, cell migration, and tumor suppression (18, 51). They consist of seven soluble proteins, designated by the letters A to G, and are secreted by different cell lineages, such as neurons, epithelial cells, or tumor cells (51). Each of the SEMA 3 member has different binding affinities to NRP1, and therefore each SEMA 3 member has a distinct biological function. The binding of the SEMA to NRP1 is established through the a1, a2, and b1 domains (19, 51). This binding requires an association with another family of receptors: the plexins. Indeed, by acting as a co-receptor, NRP1 increases the binding affinity of plexins to SEMA 3. The tri-complex formed by NRP1, SEMAs, and the plexins enhances signal transductions during embryonic development, axon guidance, and immune responses. For example, the interaction between NRP1 and Sema3E/PlexinD1 transforms axonal repulsion into attraction during brain development (57). NRP1 has been found to bind preferentially to SEMA3A, while NRP2 usually binds to SEMA3C or to SEMA 3F (3, 51).

#### VEGF and VEGFRs

In addition to binding to SEMA, NRP1 is now known to be a receptor for the VEGF family (10). The VEGF family consists of several growth factors, designated A, B, C, and D, as well as placental growth factor (PlGF), whose main role is to mediate angiogenesis and lymphangiogenesis. The classical VEGF receptors are tyrosine kinase receptors called VEGFR-1, VEGFR-2, and VEGFR-3. These three VEGFR have different affinities with VEGF ligands and thereby different biological actions. For example, VEGFR-2, the most known VEGF receptor, binds preferentially to VEGF-A, is expressed mainly by vascular endothelial cells and therefore plays a key role in vasculogenesis and angiogenesis (50). NRP1 was initially identified as a specific receptor for the VEGF-A 165 isoform (10), and more recently, for other isoforms, such as VEGF-A 121 (58). It is the extracellular b1b2 domains of NRPs that are involved in this binding (59). NRP1 seems to act mainly as a co-receptor for VEGFR-1 and -2, an important actor of the angiogenesis, whereas NRP2 is a co-receptor for VEGFR-3, a critical receptor of lymphangiogenesis (50).

#### Other Ligands of NRP1

NRP1 interacts with other proteins, such as the fibroblast growth factor (FGF) (60), the hepatocyte growth factor (HGF) (61, 62), the platelet-derived growth factor (PDGF) (63, 64) and the transforming growth factor β (TGF-β) (65, 66).

According to the studies of West et al., NRP1 binds to FGF isoforms (FGF-2, 4, and 7) and to their receptor FGFR-1, thus contributing to angiogenesis. The same team also showed that NRP1 binds to HGF, which is involved in various functions, such as cell proliferation, cell migration, and morphogenesis (60, 61). The binding of NRP1 to HGF potentiates the activity of the growth factor with its receptor, c-MET, which promotes cell survival and invasiveness. This potentiation of HGF activity by NRP1 contributes to the development of certain cancers, such as gliomas and pancreatic ductal adenocarcinomas (61, 62). Moreover, the discovery of NRP1 binding to PDGF confers a new role to NRPs in the regulation of PDGF signaling within vascular smooth muscle cells. NRP1 and NRP2 are highly expressed in vascular smooth muscle cells, and their association with PDGF plays a role in the migration, proliferation, and survival of these cells (66), as well as in other cell types, such as in mesenchymal stem cells (67).

Other studies have described that NRP1 plays a critical role in vascular development and homeostasis *via* the regulation of TGF-β in endothelial cells (65, 66, 68). Indeed, NRP1 binds to TGF-β in its active and latent forms associated with the LAP protein (LAP-TGF-β) *via* the b1 domain. Moreover, these two forms of TGF-β compete with VEGF 165 for binding to NRP1 (65). In addition to binding to TGF-β, NRP1 can form complexes with all three forms of TGF-β receptors (TGFβR), but has a higher affinity for TGFβR-1 (68). Another study showed that NRP1 in endothelial cells suppresses TGF-β activation and signaling by forming protein complexes with the integrin β8, suggesting an inhibitory role for NRP1 on TGF-β signaling (69).

### Involvement of Neuropilin-1 in Pediatric Brain Cancers

The ability of NRP1 to bind to a variety of ligands and receptors involved in different signaling pathways, such as SEMA 3 or VEGF for example, suggests that NRPs are involved in many physiological and pathophysiological processes, such as cancer. Several studies have shown that NRP1 is expressed in tumor cells of many cancers, such as breast cancers (16), lung cancers (70), pancreatic cancers (15), oral cancers (71), or brain tumors, such as gliomas (12, 72) and medulloblastomas (14). Moreover, the overexpression of NRP1 in tumors is associated with poor prognosis for those patients. Indeed, several research teams have noted that, when NRP1 is overexpressed in tumor tissues, such as pediatric brain tumors, the prognosis for those patients was poor in contrast to the patients without an overexpression of the receptor. Therefore, NRP1 overexpression is described as a poor prognostic factor in various cancers, such as lung cancers (70), pancreatic cancers (15), liver cancers (13), breast cancers (16), gliomas (12), and medulloblastomas (14).

**Figure 2:**
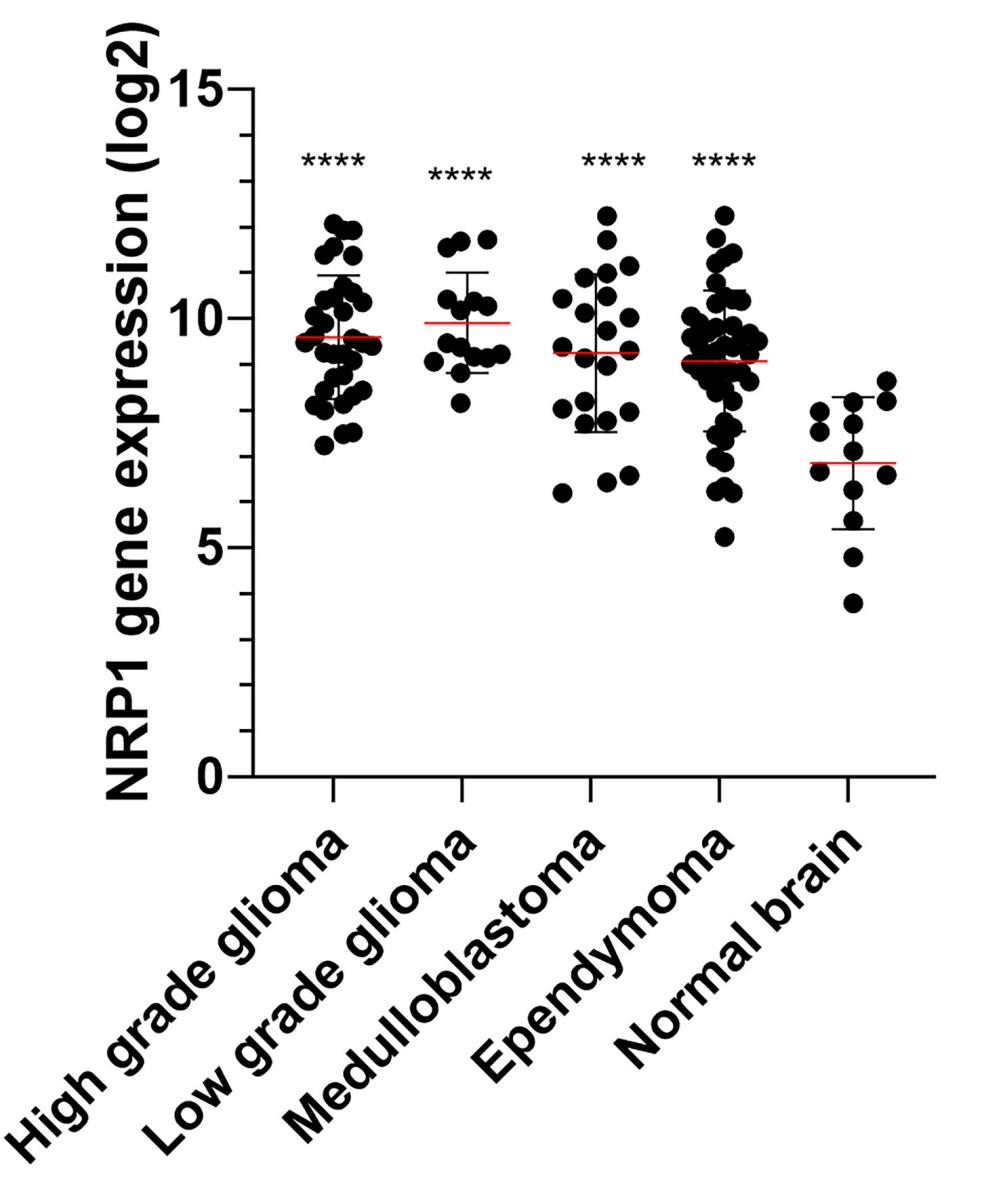
NRP1 expression (Log2) in normal brain and pediatric brain tumor samples. Microarray (Affymetrix HG-U133 plus 2) expression of 13 normal, 34 high grade glioma, 15 low grade glioma, 22 medulloblatoma and 46 ependymoma were extracted from the GSE50161 dataset (http://www.ncbi.nlm.nih.gov/geo/query/acc.cgi?acc=GSE50161). The significance was evaluated by a one-way ANOVA followed by a post-hoc Tukey for multiple comparisons. ****P < 0.001 *versus* normal brain.

Furthermore, the poor prognosis of patients with NRP1 overexpression in their tumor cells is due to the involvement of NRP1 in tumor progression and tumor growth. Several studies have highlighted the link between NRPs and tumor progression in different cancer types, notably in brain cancers. More precisely, NRP1 was expressed in different cerebral tumors at a relatively high level of expression, no matter the type or the grade of the tumor (**Figure 2**), while the expression of NRP2 seems to be constantly low in all brain tumors (73, 74), demonstrating that its presence alone is correlated with poor prognosis (75). Thus, NRPs have been shown to play an important role in tumorigenesis, tumor development, tumor invasion, and tumor angiogenesis in adult brain tumors.

NRP1-mediated tumor invasion and growth has been described in several publications studying gliomas and glioblastomas. NRP1 appears to influence the tumor cell proliferation and migration by interacting with various proteins and signaling pathways. Indeed, Zhang’s team demonstrated that NRP1 mediates glioma progression through its interaction with the intracellular protein GIPC1 *via* the cytoplasmic C-terminal SEA motif (76). Inhibition of GIPC1 decreases glioma cell proliferation and invasion and increases apoptosis *in vitro* (76). NRP1/GIPC1-mediated proliferation, angiogenesis, and migration appear to be related to the activation of PI3K/Akt/NFkB signaling pathways (77). Another study suggested that NRP1, in combination with Plexin-A1, was correlated with poor prognosis in glioblastomas and may contribute to tumor growth. A peptide, specifically targeting Plexin-A1, has shown promising results in reducing proliferation and angiogenesis as well as blocking tumor cell spread following disruption of NRP1 and Plexin-A1 heterodimerization (78). On the other hand, some current treatments seem to have an impact on the expression of NRP1 and inversely. The use of temozolomide (TMZ) in combination with inhibition of VEGFR signaling appears to have beneficial effects in the treatment of glioblastomas as TMZ has been shown to reduce the expression of NRP1 and thus induce an increase in treatment cytotoxicity (79). More recently, a study of patient-derived glioblastoma multiforme xenografts in zebrafish and mouse models have reported that depletion of NRP1 inhibits the growth of the tumor and substantially prolongs the survival rate of mice in comparison with VEGF-depletion, by improving sensitivity to TMZ (80). This work has shown that the ablation of NRP1 appears to improve the therapeutic response in glioblastomas (80). NRP1 appears to be a potential therapeutic target, especially as it is involved in the recurrence of tumors, such as GBM, by modulating TGF-β signaling after anti-angiogenic therapies. NRP1 regulates GBM growth and invasion by balancing tumor cell responses to VEGF-A and TGF-β (81). GBMs that recur after bevacizumab treatment show down-regulation of NRP1 expression. The altered balance between VEGF-A and TGF-β signaling mediated by NRP1 is a mechanism that promotes resistance to anti-angiogenic agents (81).

Unlike adult brain tumors, studies in pediatric brain tumors mainly focus on medulloblastomas. NRPs are overexpressed in MB and correlated with poor prognostic (14). Several studies show that targeting NRP1 reduces MB. Indeed, Snuderl et al. obtained MB regression in an intracerebral xenografted mouse model by targeting the PlGF/NRP1 signaling pathway. Inhibition of PlGF/NRP1 decreases tumor growth and metastasis in mice, and increases their survival rate (14). Recent studies have shown the benefit of NRP1 inhibition to decrease MB progression (82, 83). Indeed, Gong et al. show that inhibition of NRP1 prevents the invasion and tumorigenicity of MB cells. This phenomenon is linked to decrease activation of the PI3K/Akt and MAPK pathway *in vitro* in the Daoy MB cell line (82). Furthermore, NRP1 also appears to be implicated in treatment resistance of pediatric brain tumors. Researchers have shown that targeting 4D phosphodiesterase, interacting directly with NRP1, inhibits tumor growth *in vivo*, even in mice resistant to vismodegib, an anti-SMO treatment (84). Concerning NRP2, few studies have described its involvement in pediatric brain tumors. It seems to be particularly implicated in SHH subgroup of MBs, because NRPs are involved in Hh signal transduction. This study shows that a knockdown of NRP2 has more anti-tumor effects than a knockdown of NRP1, as loss of NRP2 decreases cell proliferation while loss of NRP1 influences cell migration (85).

Intriguingly, the findings of Ishizuka et al. suggest that NRP1 has a tumor suppressive effect on neuroblastomas, a childhood extra-cranial solid malignant tumor (86). In neuroblastomas, a higher level of NRP1 expression was associated with a longer survival time (86). These findings are in contradiction with the findings of the previous reports on NRP1 in other cancer types described previously in this review.

As detailed above, NRP1 is involved in tumorigenesis in multiple ways, as it is played a role in different signaling pathways. Its best known involvement is in angiogenesis (18, 19), but this review focuses on the role of NRP1 in cancer stem cells (CSC) and in immune system (SI) during brain cancers development.

## Neuropilin-1 and Brain Tumor Stem Cells

### Cancer Stem Cells in Brain Tumor

Tumor recurrence and the spread of metastases are among the major challenges in the treatment of brain tumors. These events are complicated by the heterogeneity of tumor cells present in all solid tumors, such as brain tumors. Solid cancers are organized in a hierarchical manner, it is suggested that a small number of cells are able to drive tumor growth, this population of cells is called CSCs or initiating cells tumor. Indeed, the CSC hypothesis is that not all the cells in the tumor have the same ability to proliferate and to maintain the growth of the tumor. There are two theoretical models to explain the presence of these CSCs in tumors: the stochastic model, where each cancer cell has the capacity to dedifferentiate into a CSC, and the hierarchical model where CSCs are considered the progenitors of differentiated cancer cells, with the capacity of self-renewal, differentiating, and expanding the pool of CSCs (87–89).

Furthermore, secondary resistances almost constantly occur in cancer, and CSC are suggested as a potential source of this chemoresistance and a lower survival rate. Indeed, CSCs survive chemo-radiotherapy and therefore contribute to multiple drug resistance mechanisms (90). Many mechanisms have been suggested for CSC resistance, such as drug efflux through ABC transporters, over-activation of the DNA damage response, apoptosis evasion, pro-survival pathways activation, cell cycle promotion, and/or cell metabolic alterations (91, 92). CSCs have also been found to be associated with tumor dissemination through the epithelial-mesenchymal transition (EMT), a biological process which increases the invasion and the motility of cancer cells that are essential for distant colonization (93). These CSCs thus contribute to the poor prognosis of cancers (88, 89). CSCs have been characterized in multiple cancer types, including breast (94), colon (95), brain (96), and ovarian cancers (97).

Multiple pediatric brain tumors have been reported to harbor CSCs, including MBs (98, 99) and gliomas (99, 100). Most reports have identified CSCs by isolating the cells which are able to self-renew, differentiate, re-form the initial tumor, and present stem markers (99). In addition, those properties must be demonstrated *in vitro*, but the CSCs must also present the capacity to develop a tumor *in vivo*. In a SHH MB model, Zhang et al. unexpectedly found by using single-cell transcriptomics that progenitors of the glial lineage are the cells that propagate rapidly during the initial phase of tumorigenesis, although in complete tumors these cells are quiescent and have stem-like properties. Moreover, these authors showed that MB progenitors are enriched in recurrent and treatment-resistant tumors to cisplatin and cyclophosphamide and may serve as a niche for tumor initiation (101).

Great advances in sorting and identifying CSCs in brain tumors by using various markers have been made during the last decade. The first marker of CSCs in brain tumors was described in a study by Singh et al. (98). They have identified a new population of CSCs: the brain tumor cancer stem cells (BTSC). These cells express the neural stem cell marker CD133, do not present the differentiated cell markers and have the stem cell properties *in vitro*. The study showed that the CD133 cells have the capacity to form cell clusters derived from clones, such as neurospheres. BTSC renew themselves, proliferate, and differentiate by reproducing the phenotype of the original tumor (98). Their research team have published data showing that BTSC have the capacity to initiate the tumor *in vivo* (100) and further exploration have shown that CD133+ cells are able to initiate a new tumor in immunodeficient mice, whereas CD133− cells could not (98). Nevertheless, CD133 seems to be not an ideal cancer stem cell marker for pediatric brain tumors. Since then, mouse models have been developed that can classify pediatric brain tumor CSCs based on their expression of CD15 (102), Nestin (103), or Sox2 (104). CD15 (FUT4, SSEA-1) has shown a particular interest as a cancer stem cells marker for pediatric brain tumors as HGGs and LGGs (105) or MBs (106).

### NRP1 Signaling Pathway in CSC

**Figure 3:**
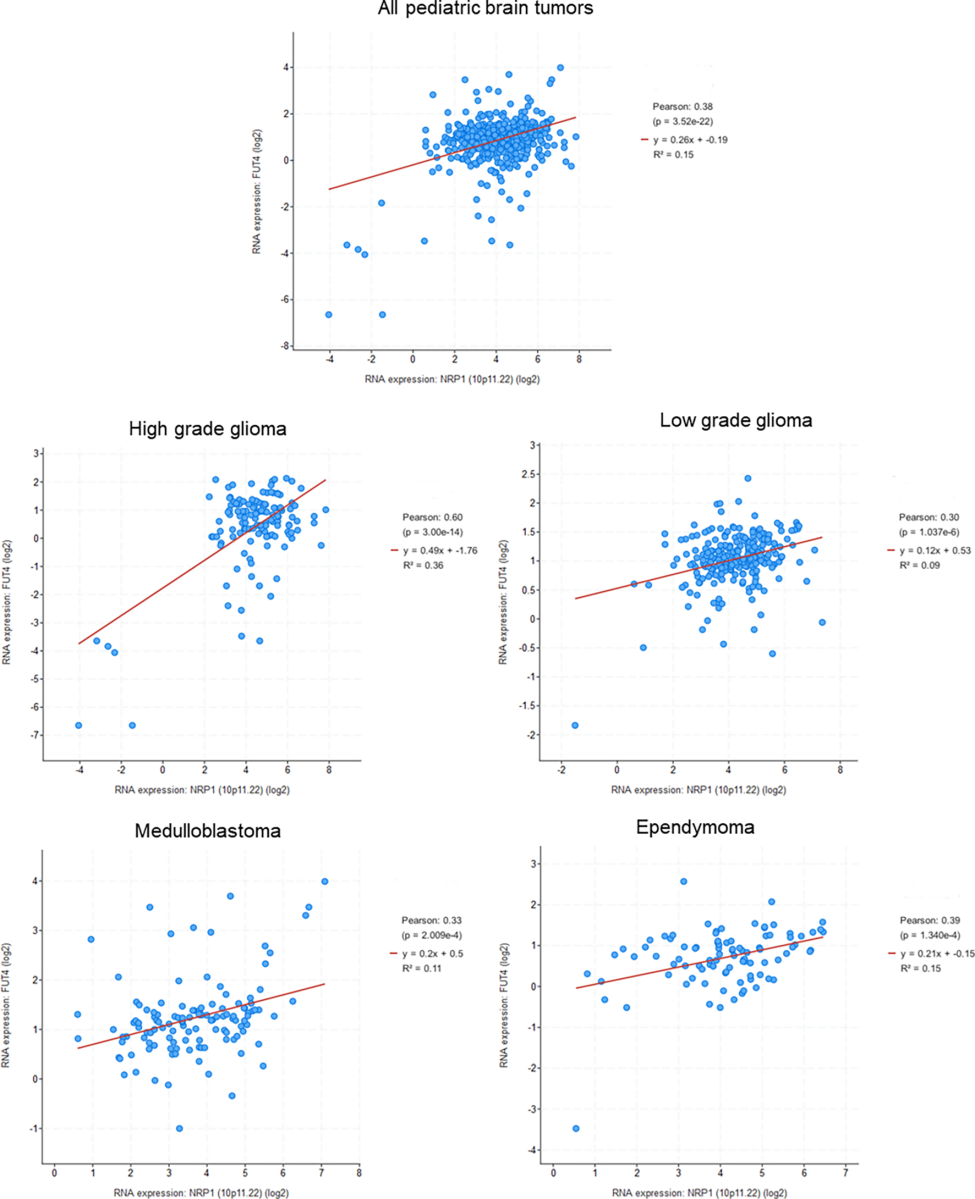
Correlation between NRP1 and CD15 (FUT4) expression in pediatric brain tumors (Genomic characterization has obtained with cBioPortal provided by the Children’s Brain Tumor Tissue Consortium Database; Pediatric High Grade Gliomas: 133 samples; Pediatric Low Grade Gliomas: 261 samples; Medulloblastoma: 130 samples; Ependymomal Tumor: 97 samples). Pearson's correlation coefficient and corresponding p value were given for each tumor types.

Several studies have shown that NRPs signaling promotes CSC survival and tumor aggressiveness (55, 88, 107, 108). Because of their difference in expression, NRP1 and NRP2 are probably different in their ability to promote the functionality of CSCs. Moreover, it seems that they also differ in their signaling properties. Indeed, Grun et al. found that the VEGF/NRP1 signaling pathway was involved in the survival of epidermal cancer stem cells (ECS), contributing to tumor aggressiveness (55). VEGF-A is required to maintain the ECS phenotype and this process does not involve the classical VEGF receptors, but NRP1. Inhibition of NRP1 expression in ECSs reduces spheroids formation, their invasion and migration, and thus decreases tumor development (55). This has been described as well as for breast cancer CSCs (107, 108). Breast cancer CSCs overexpress VEGF-A and NRP1. Zhang et al. demonstrated, using a CSC KO model for NRP1 or VEGF-A, that this signaling axis was necessary to ensure the specific traits of CSCs *in vitro* and *in vivo*, and that the VEGF-A/NRP1 axis conferred the strain phenotype to breast cancer cells by activating the WNT/β-catenin signaling pathway (108). In the same way, Liu et al. have shown that NRP1 expression is induced by Wnt/β-catenin signaling in mammary stem cells, and its suppression leads to decreased tumorigenesis *in vivo* models (107). Furthermore, analysis of pediatric brain tumors databases shows a significant correlation between NRP1 and CD15 for the four analyzed tumor types. This correlation seems especially high for high grade gliomas, and so reinforces the possible role of NRP1 in tumor progression (**Figure 3**).

However, it appears that the interaction between NRP1 and VEGF is not the only factor of survival for CSCs. Indeed, some studies have shown that the interaction between NRP1 and GIPC1 is also involved in this phenomenon. In epidermal stem cells, NRP1 forms a complex with GIPC-1 and the integrin α6/β4 to activate the FAK/Src signaling pathway that will then allow the stabilization of YAP1/ΔNp63α which, consequently, leads to enhanced ECS survival, invasion, and angiogenesis (109). NRP1/GIPC1 is also a key signal in the deformation character of ECS when it forms a complex with Syx, the guanine-RhoA exchange factor involved in angiogenesis. This interaction improves the spheroid formation, invasion, migration, and angiogenic potential of the cells *in vitro*. The formation of this complex leads to an increase in the activity of the p38 MAPK pathway, which is dependent on RhoA (109). In combination, when NRP1 or GIPC-1 expression is suppressed, both RhoA and p38 MAPK activity is decreased, leading to the loss of the ECS phenotype and reduce tumor growth (109).

Some studies also attest that NRP2 contributes to CSC function and tumor formation. Its involvement in CSC has been mainly described in breast cancers (110, 111). NRP2 is expressed in CSC of breast cancers and Goel et al. have described that VEGF/NRP2 signaling is important in tumor initiation. Indeed, NRP2 maintains the tumor-initiating cells by stimulating α6β1 integrin, this interaction inducing the FAK/Ras pathway and thus leading to the activation of GLI1. GLI1 also induces BMI-1, a key stem cells factor, which enhances NRP2 expression (111). Another study demonstrates that VEGF/NRP2 signaling pathway contributes to the mammosphere formation (110). This occurs through the inhibition of Rac GTPase-activating protein β2 chimaerin mediated by activation of TAZ, a terminal effector of Hippo signaling associated to breast cancer stem cells (110). This result shows that NRP2 also plays a role in the acquisition of CSC properties in breast cancers (110).

Thirant et al. have isolated cells with the same properties as stem cells in different brain tumors that were associated with poor prognosis. In adult brain tumors, these stem-like cells were isolated only in HGGs. In contrast, stem-like cells have been isolated from different subtypes and grades in pediatric brain tumors (112). Several studies have therefore focused on the function and the target of these brain cancer stem cells *via* NRPs. Hamerlik et al., working on gliomas, have highlighted that the viability and self-renewal of stem cells, and therefore their tumorigenicity, relies on the VEGF/VEGFR2/NRP1 signaling pathway (72). Their results suggest that inhibiting VEGFR2 or NRP1 in these cells could be a safe therapy in contrast to bevacizumab, a monoclonal antibody targeting VEGF, which remains ineffective against glioma CSCs (72). Recently, a study on glioblastomas has shown that Sema 3C, secreted by glioblastoma stem cells, forms a complex with PlexinA2/D1 and NRP1, promoting glioblastoma self-renewal and sphere formation. This complex is only functional in presence of NRP1 and allows the activation of Rac 1, which is an actor of tumor cells survival (113).

Currently, few studies have investigated the relationship between NRPs and CSCs in pediatric brain tumors. A study conducted by Gong et al. found that NRP1 plays a key role in the undifferentiated phenotype of MB CSC (82). First, they note that NRP1 is over-expressed in MB CSC. Secondly, their results show that, by inhibiting NRP1 *via* a peptidomimetic specifically targeting the protein, stem cells lose their stem characteristics, such as Sox 2 or CD15. Finally, the loss of NRP1 expression decreases the invasiveness capacity of MB CSC. This study suggests that the use of a molecule targeting NRP1 may have a relevant to target CSC in the case of MBs. This targeting of CSC could prove to be even more effective on survival rates, since several studies have shown that CSC are involved in the dissemination of metastases and tumor recurrence (114). Garg et al. have shown that MBSC CD133+ cells are associated with an increased metastasis and poor clinical outcome (115).

## Neuropilin-1 And The Immune System Of Pediatric Brain Tumors

### NRP1 Plays a Major Role in the Immune System

Interestingly, NRP1 also plays an important role in cellular immunity, whether in physiological or pathophysiological conditions, such as cancer. However, currently little knowledge of the molecular pathways involved in these functions. The expression of NRP1 has been characterized in different immune cell types, such as macrophages, dendritic cells (DCs), and lymphocytes, in particular regulatory T lymphocytes (LTreg) (116–121) and the NRP1 involvement in immune responses to cancer is summarized in **Table 1**.

**Table 1:** Summary NRP1 involvement in the immune system.

Expression	Effects	Signaling pathway	Related disease	Reference
**Dentritic cells (DC)**	• Migration	• SEMA3A/SEMA3C/PlexinA1		(118, 122)
	• Reorganization of F-actin cytoskeleton			(118)
	• Recognition of pathogenic antigens,			(123, 124*)
	Formation of immunological synapse with T cells			
	by interaction between NRP1 on DC and T cells			
	• NRP1 transfer from DC to LT	• VEGF-A		(125)
**Macrophages/microglia**	• Promotion of macrophages type M2	• SEMA 3A	• Cancer	(119, 121*, 126)
	• Migration tumor-associated macrophages (TAM)	• SEMA 3A	• Cancer	(127*)
** **	• Infiltration within tumor	• SEMA 3A	• Cancer	(127*, 128–130)
**Lymphocytes**				
• Thymocytes	• Decrease adhesion capacity,	• SEMA 3A		(131, 132)
	Migration by repulsive effect			
• Lymphoctes T CD4+	• Supression of T cell proliferation and		• Autoimmune disease	(117, 133*)
	their cytokines production			
• Lymphocytes T CD8+	• Inhibition of migration within tumor,	• SEMA 3A	• Cancer	(134, 135)
	Inhibition of tumor cell lyse function			
• Lymphocytes Treg	• Immunosuppression and induction of tolerance	• SEMA 4A/PlexinA4,		(136–138*)
	• Increase the number of LT cells,	B7-H4/SEMA 3A/Plexin A4		(136, 137)
	Increase of IL10 secretion			
	• Prolongation of interaction between LTreg and DC			(124*)
	• Stability and function of LTreg	• SEMA 4A		(136, 137, 139)
	• Infiltration of LTreg within tumor,		• Cancer	(138*)
	and tumor immune escape function			
	• Treg activation	• VEGF-A	• Cancer	(140)
	and increase of TGFβ production by LTreg			

*References with a murine model.

Some research teams working on autoimmune diseases have shown that NRP1 plays a critical role in the regulation of immune responses. Solomon et al. investigated multiple sclerosis with an autoimmune encephalomyelitis (EAE) model of mice deficient in NRP1 on their T CD4+ lymphocytes (LT CD4+) and showed that mice presented aggravated EAE in contrast to mice over-expressing NRP1 on their LT CD4+, which did not develop EAE (133). This suggests an anti-inflammatory role of NRP1, which has also been highlighted in a model of rheumatoid arthritis *via* the SEMA 3A/NRP1 signaling pathway (117). We know that NRP1 is a functional receptor for semaphorins and is expressed on LTs (117, 131). Although NRP1 is weakly expressed on Tregs isolated from human blood and cannot be used as an identifying marker for circulating human Tregs, it has recently been shown that in humans, NRP1 is expressed on CD4+ tumor-inflitrating lymphocytes (TILs), including Tregs (141). Furthermore, SEMA 4A/Plexin A4/NRP1 signaling has been implicated in the increased number of LTreg cells that are essential for the maintenance of peripheral tolerance and regulation of immune responses, and in the potentiation of their function. Moreover, the formation of the SEMA 4A/Plexin A4/NRP1 complex is linked to an increase in the secretion of interleukin 10 (IL10), an anti-inflammatory interleukin, thus contributing to the regulation of the immune response (136). More recently, it has been shown that the B7-H4 ig protein binds to the SEMA 3A/Plexin A4/NRP1 complex to modulate the Treg response. Since the B7-H4ig protein is associated with the regulation of immunity by reducing CD4+ LT inflammatory responses, this suggests an immune-regulatory role for the SEMA A3/Plexin 4A/NRP1 complex (137). NRP2 is also expressed in T cells (120, 132), and it has been shown that NRP2/plexinA1 interacts with Sema 3F to inhibit mTOR and PI3K signaling in T cells (120). Furthermore, NRP2 co-immunoprecipitated with PTEN, a pro-tumor protein, required by SEMA 3F for the inhibition of mTOR and PI3K pathway (120).

In addition to playing an immune-regulatory role, NRPs are also involved in the immune cell migration. For example, a study showed that SEMA 3A decreases the adhesion capacity of thymocytes expressing NRP1 and induces their migration by a repulsive effect (131). In the same way, the SEMA 3F/NRP2 pathway controls the migration of human T cell precursors (132). In addition, NRP1 and NRP2 are expressed by dendritic cells and play a role in their migration (118, 122). NRP1 forms a complex with Plexin A1, which is known to play a crucial role in DC trafficking. The formation of this complex on the surface of DCs allows the binding of SEMA 3A present in the lymphatic vessels, which then leads to the migration of DCs into the lymphatics (122). Curreli et al. showed that SEMA 3A, 3C, and 3F promote DCs migration and that the function of SEMA 3F on DC migration is related to this binding of NRP2 (118).

Importantly, NRP1 is expressed on the surface of DCs involved in an important step for the initiation of immunity: the recognition of pathogenic antigens. Indeed, DCs are antigen-presenting cells (APCs), which have the ability, *via* MHC-I and MHC-II molecules, to present an antigen. APCs present antigen by forming an immunological synapse with LTs and thus stimulate their responses. NRP1 is expressed on both the surface of CDs and LTs and appears essential in their interaction to initiate the immune response (123). In particular, in murine model, NRP1 is expressed by LTreg, which improves and prolongs their interaction with DCs during the immunological synapse, thus conferring them a higher sensitivity to antigens (124). Moreover, another study points to NRP1 may be transferred from DCs to LTs within this immunological synapse, allowing them to bind to VEGF-165. These results suggest that NRP1-expressing LTs may be involved in vessel remodeling in secondary lymphoid organs during inflammation (125).

Macrophages, another important player in immunity, also express NRPs (121, 126). They are immune cells present in all tissues and play a role in immune surveillance, inflammation response and resolution, and also contribute to wound healing. They originate from monocytes and NRP1 appears to play a role in their differentiation. Indeed, the expression of SEMA-3A and its receptor, NRP1, significantly increases during the monocyte differentiation into macrophages, and specifically during the monocyte differentiation under conditions favoring the macrophages of M2 phenotypes, which are characterized by anti-inflammatory and pro-angiogenic phenotype. The expression of NRP1 appears to decrease during the monocyte differentiation when conditions are in favor of macrophages with pro-inflammatory M1 phenotype (119). Macrophages also express NRP2 and it appears to be involved in monocyte differentiation into macrophages, but not to the same extent as NRP1 (119, 121).

### NRP1 Decreases Efficiency of the Immune System in Cancer

**Figure 4:**
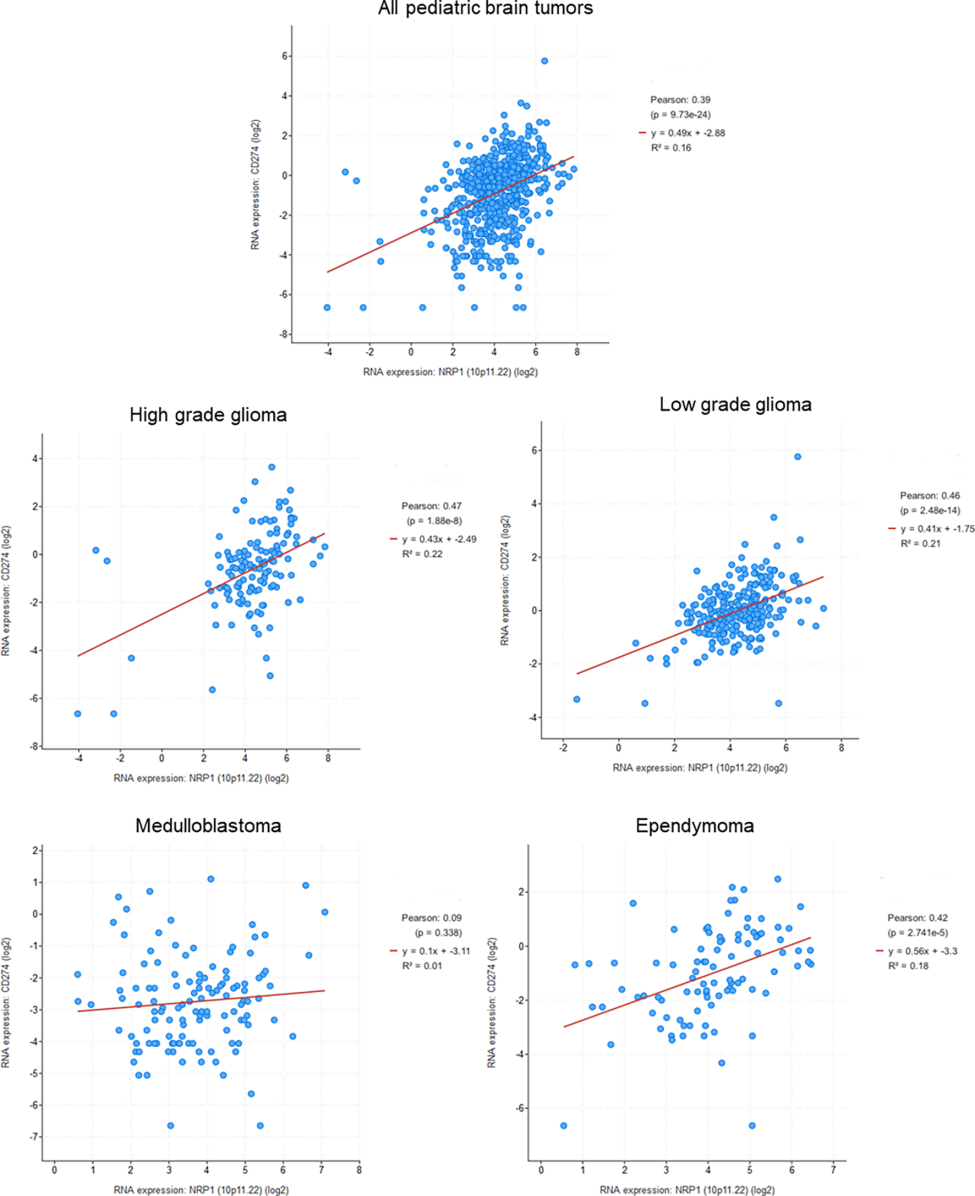
Correlation between NRP1 and PDL1 (CD274) expression in pediatric brain tumors (Genomic characterization has obtained with cBioPortal provided by the Children’s Brain Tumor Tissue Consortium Database; Pediatric High Grade Gliomas: 133 samples; Pediatric Low Grade Gliomas: 261 samples; Medulloblastoma: 130 samples; Ependymomal Tumor: 97 samples). Pearson's correlation coefficient and corresponding p value were given for each tumor types.

The immune system plays an essential role in our organism, especially during cancer when its immunological surveillance system is essential to control the tumor development and eliminate tumor cells. However, an important aspect of tumor progression is the ability of cancer cells to evade the monitoring and clearance by the immune system. Indeed, tumors cells avoid the immune system by different mechanisms, such as reducing the immune recognition, increasing the resistance to immune attacks, or creating an immunosuppressive microenvironment and recruiting specific immune cells that favor tumor growth and progression (142, 143). Because of their expression in many immune cells and its involvement in the function of immune cells, NRPs have been the subject of some studies concerning its involvement in the deregulation of anti-tumor immunity. However, many unknowns remain to be clarified, and the involvement of NRPs in the antitumor immunity seems to be unclear. MBs had the lowest amount of PD-L1 and cytotoxic lymphocytes of all pediatric brain tumors and, overall, a very small amount of infiltrating immune cells (144). This suggests that the tumor either actively modulates the immune response or simply has little immunogenicity, as it is suggested by the relatively low mutational burden (144). Analysis of pediatric brain tumors database shows a significant correlation between NRP1 and PDL-1 for three tumor types, such as HGGs or LGGs, as well as EPNs, and a weak correlation for MBs (**Figure 4**). This correlation highlights the possible role of NRP1 in the regulation of tumor immune system.

In the context of cancer, LTreg infiltration is a critical step in tumor development and progression. NRP1 is expressed by LTreg and promotes their immunosuppressive effect by at least two pathways. First, NRP1, by acting as a VEGF co-receptor, will guide the LTreg within the tumor. Data from Hansen et al. show, in a mouse model of melanomas, that this infiltration of LTreg through NRP1 induces a decrease of the anti-tumor immune response and an improvement in tumor progression (138). In a second step, NRP1 maintains the stability and intratumoral function of LTreg cells *via* the SEMA-4A signaling pathway (136), and the inhibition of NRP1 in intratumoral LTregs promotes the production of interferon-γ (IFNγ), which leads to the fragility of surrounding LTregs, thereby enhancing anti-tumor immunity and facilitating tumor clearance (139).

Recently, NRP1 was found to modulate tumor-specific CD8+ T lymphocytes (LT CD8+) cell responses (134, 135). Indeed, Leclerc et al. demonstrated that NRP1 interacts with SEMA 3A by inhibiting the migration of cytotoxic LT CD8+ within the tumor and inhibiting their tumor cell lysis function (135). Data show that neutralizing NRP1 with a specific antibody improves the migration of LT CD8+ infiltrating the tumor and their cytotoxicity (135). This finding with NRP1 neutralization in an *in vivo* melanoma model would allow for synergistic action with anti-PD1 therapies in controlling tumor growth (135). However, the role of NRP1 on LT CD8+ is still unclear and controversial. Another study has shown that NRP1 allows cross-presentation: a necessary process against tumors allowing the recognition and destruction of tumor cells by LT CD8+ (134). Indeed, NRP1, expressed on the surface of breast cancer tumor cells, allows the internalization of neutrophil elastase, which results in the cross-presentation of the PR1 antigen and which is necessary for the specific lysis of tumor cells by cytotoxic T lymphocytes (134).

Another immune cell involved in anti-tumor immunity is the macrophage. Tumor-associated macrophages (TAMs) are strongly associated with tumorigenicity. NRP1, *via* its interaction with SEMA 3A, is involved in their migration. Indeed, the work of Casazza et al. shows that NRP1 controls the recruitment of TAMs within tumors. Its inhibition within macrophages causes a reduction of their pro-angiogenic and immunosuppressive effects and leads to an inhibition of tumor growth and metastasis (127). A recent study demonstrates another mechanism of action of NRPs on macrophages in a tumor context. Thus, NRP2, expressed during macrophage differentiation, is induced by tumor cells and regulates macrophage phagocytosis. NRP2 promotes efferocytosis in macrophages, a phagocytosis that eliminates apoptotic cells in an immunologically silent manner (128). In this study, NRP2 inhibition, in a pancreatic cancer model, results in delayed clearance of apoptotic cells, and lead to increased LT CD8+ and NK cell infiltration within the tumor, consequently decreased in tumor growth. Inhibition of NRP2 may have a direct effect on the ability of TAMs to express their immunosuppressive functions *via* the expression of IL10 or TGF-β for example (128).

NRPs are, therefore, involved in anti-tumor immunity and it is questionable whether the same is true for brain tumors. The central nervous system was classically considered as an “immune-privileged site,” being immunologically inert and separated from the peripheral immune system by the blood-brain barrier (BBB). However, brain tumors represent a particular case in which the BBB is disrupted to various degrees, and therefore, acquired an innate immunity that may play a role in the development and growth of brain cancers. Most studies have focused on adult gliomas, and very few so far have focused on pediatric brain tumors. Several studies led by Miyauchi et al. have shown that NRP1 is expressed by macrophages and microglia associated with gliomas (129, 130). In their NRP1-deficient mouse model, they suggest that the loss of NRP1 expression by macrophages and/or microglia is associated with delayed tumor growth (129, 130). Another study describes the role of NRP1 on LTreg in glioma and shows that a small molecule antagonist of NRP1 is able to block a glioma-conditioned medium-induced increase in TGF-β production in LTreg (140).

## Conclusion

NRP1 were known since the end of the 1990s to play a role in axonal guidance and vascular biology, acting as a co-receptor for plexin and VEGF receptors families respectively. Furthermore, this co-receptor is also expressed by tumor cells and may be involved in the progression of tumors. In this review, we have discussed the involvement of NRP1 in pediatric brain tumors, such as gliomas, MBs, or EPNs. Indeed, the NRP1 signaling pathway is clearly involved in the cancer stem cells maintenance in pediatric tumors, and a significant correlation between NRP1 and CD15 (stem cancer cells marker) has been observed in pediatric tumors database. Moreover, NRP1 is involved in the activation of the immune cells, in particular in the interaction between T cells and DCs. NRP1 may also play a role in the regulation of immunogenicity of tumor cells, highlighted by the correlation between NRP1 and PDL-1. This role is not yet fully investigated, but we will focus our future research on this aspect. Numerous peptides or biological molecules that target NRP1 and whose efficacy has been tested *in vitro* and *in vivo* have been developed (19). For the moment, only clinical phase I trials have been performed against NRP1 with monoclonal antibodies. The first has been performed with MNRP-1685A for patients with solid tumors (145) and more recently, with TB-403, targeting the PlGF-NRP-1 axis, in pediatric subjects with relapsed MB (146). To conclude, NRP1, through its different action ways, could be a key protein in the progression of pediatric brain cancers, and could be envisaged as a therapeutic target for these tumors.

## Author Contributions

MD, PC, and CB contributed to redaction of this review. MD wrote the first draft of the manuscript. All authors contributed to the article and approved the submitted version.

## Conflict of Interest

The authors declare that the research was conducted in the absence of any commercial or financial relationships that could be construed as a potential conflict of interest.

## Abbreviations

APC, antigen-presenting cells; BBB, blood-brain barrier; BTSC, brain tumor cancer stem cells; CSC, cancer stem cells; CNS, central nervous system; DC, dendritic cells; DIPG, diffuse intrinsic pontine glioma; EAE, autoimmune encephalomyelitis; ECS, epidermal cancer stem cells; EMT, epithelial-mesenchymal transition; EPN, ependymoma; FGF, fibroblast growth factor; GIPC1, GAIP-interacting protein C terminus member 1; HGF, hepatocyte growth factor; HGG, high-grade glioma; IL10, interleukin 10; K27M, mutations at position 27; LGG, low-grade glioma; LTreg, regulatory T lymphocytes; LT CD4+, T CD4+ lymphocyte; LT CD8+, T CD8+ lymphocyte; MAPK, mitogen-activated protein kinase; MB, medulloblastoma; NRP, neuropilin; NRP1, neuropilin-1; NRP2, neuropilin-2; PA, pilocytic astrocytoma; PDGF, platelet-derived growth factor; PFS, progression-free survival; PlGF, placental growth factor; PMA pilomyxoid astrocytoma; PXA, pleomorphic xanthoastrocytoma; SEGA, subependymal giant cell astrocytoma; SEMA 3, semaphorin of class 3; SHH, sonic hedgehog; SI, immune system; TAMs, tumor-associated macrophages; TGF-β, transforming growth factor β; TGFβR, receptors of TGF-β; TMZ, temozolomide; VEGF, vascular endothelial growth factor; VEGF-R, receptor of vascular endothelial growth factor; WHO, World Health Organization.

## References

[1] Kitsukawa T Shimono A Kawakami A Kondoh H Fujisawa H. Overexpression of a Membrane Protein, Neuropilin, in Chimeric Mice Causes Anomalies in the Cardiovascular System, Nervous System and Limbs. Dev Camb Engl (1995) 121(12):4309–18. 10.1242/dev.121.12.4309 8575331

[2] Kawasaki T Kitsukawa T Bekku Y Matsuda Y Sanbo M Yagi T. A Requirement for Neuropilin-1 in Embryonic Vessel Formation. Dev Camb Engl (1999) 126(21):4895–902. 10.1242/dev.126.21.4895 10518505

[3] Roy S Bag AK Singh RK Talmadge JE Batra SK Datta K. Multifaceted Role of Neuropilins in the Immune System: Potential Targets for Immunotherapy. Front Immunol (2017) 8:1228. 10.3389/fimmu.2017.01228 29067024 PMC5641316

[4] Lampropoulou A Ruhrberg C. Neuropilin Regulation of Angiogenesis. Biochem Soc Trans (2014) 42(6):1623–8. 10.1042/BST20140244 25399580

[5] Plein A Fantin A Ruhrberg C. Neuropilin Regulation of Angiogenesis, Arteriogenesis, and Vascular Permeability. Microcirc N Y N 1994 (2014) 21(4):315–23. 10.1111/micc.12124 PMC4230468 24521511

[6] Chaudhary B Khaled YS Ammori BJ Elkord E. Neuropilin 1: Function and Therapeutic Potential in Cancer. Cancer Immunol Immunother CII (2014) 63(2):81–99. 10.1007/s00262-013-1500-0 24263240 PMC11028473

[7] Ellis LM. The Role of Neuropilins in Cancer. Mol Cancer Ther (2006) 5(5):1099–107. 10.1158/1535-7163.MCT-05-0538 16731741

[8] Takagi S Tsuji T Amagai T Takamatsu T Fujisawa H. Specific Cell Surface Labels in the Visual Centers of Xenopus Laevis Tadpole Identified Using Monoclonal Antibodies. Dev Biol (1987) 122(1):90–100. 10.1016/0012-1606(87)90335-6 3297854

[9] Chen H Chédotal A He Z Goodman CS Tessier-Lavigne M. Neuropilin-2, a Novel Member of the Neuropilin Family, Is a High Affinity Receptor for the Semaphorins Sema E and Sema IV But Not Sema III. Neuron (1997) 19(3):547–59. 10.1016/s0896-6273(00)80371-2 9331348

[10] Soker S Takashima S Miao HQ Neufeld G Klagsbrun M. Neuropilin-1 Is Expressed by Endothelial and Tumor Cells as an Isoform-Specific Receptor for Vascular Endothelial Growth Factor. Cell (1998) 92(6):735–45. 10.1016/s0092-8674(00)81402-6 9529250

[11] Klagsbrun M Takashima S Mamluk R. The Role of Neuropilin in Vascular and Tumor Biology. Adv Exp Med Biol (2002) 515:33–48. 10.1007/978-1-4615-0119-0_3 12613541

[12] Osada H Tokunaga T Nishi M Hatanaka H Abe Y Tsugu A. Overexpression of the Neuropilin 1 (NRP1) Gene Correlated With Poor Prognosis in Human Glioma. Anticancer Res (2004) 24(2B):547–52. 15160992

[13] Yaqoob U Cao S Shergill U Jagavelu K Geng Z Yin M. Neuropilin-1 Stimulates Tumor Growth by Increasing Fibronectin Fibril Assembly in the Tumor Microenvironment. Cancer Res (2012) 72(16):4047–59. 10.1158/0008-5472.CAN-11-3907 PMC3421041 22738912

[14] Snuderl M Batista A Kirkpatrick ND de Almodovar CR Riedemann L Walsh EC. Targeting Placental Growth Factor/Neuropilin 1 Pathway Inhibits Growth and Spread of Medulloblastoma. Cell (2013) 152(5):1065–76. 10.1016/j.cell.2013.01.036 PMC3587980 23452854

[15] Ben Q Zheng J Fei J An W Li P Li Z. High Neuropilin 1 Expression was Associated With Angiogenesis and Poor Overall Survival in Resected Pancreatic Ductal Adenocarcinoma. Pancreas (2014) 43(5):744–9. 10.1097/MPA.0000000000000117 24632553

[16] Luo M Hou L Li J Shao S Huang S Meng D. Vegf/Nrp-1axis Promotes Progression of Breast Cancer Via Enhancement of Epithelial-Mesenchymal Transition and Activation of NF-κb and β-Catenin. Cancer Lett (2016) 373(1):1–11. 10.1016/j.canlet.2016.01.010 26805761

[17] Segal D Karajannis MA. Pediatric Brain Tumors: An Update. Curr Probl Pediatr Adolesc Health Care (2016) 46(7):242–50. 10.1016/j.cppeds.2016.04.004 27230809

[18] Niland S Eble JA. Neuropilins in the Context of Tumor Vasculature. Int J Mol Sci (2019) 20(3):639. 10.3390/ijms20030639 PMC6387129 30717262

[19] Dumond A Pagès G. Neuropilins, as Relevant Oncology Target: Their Role in the Tumoral Microenvironment. Front Cell Dev Biol (2020) 8:662. 10.3389/fcell.2020.00662 32766254 PMC7380111

[20] Graziani G Lacal PM. Neuropilin-1 as Therapeutic Target for Malignant Melanoma. Front Oncol (2015) 5:125. 10.3389/fonc.2015.00125 26090340 PMC4453476

[21] Johnson KJ Cullen J Barnholtz–Sloan JS Ostrom QT Langer CE Turner MC. Childhood Brain Tumor Epidemiology: A Brain Tumor Epidemiology Consortium Review. Cancer Epidemiol Biomark Prev Publ Am Assoc Cancer Res Cosponsored Am Soc Prev Oncol (2014) 23(12):2716–36. 10.1158/1055-9965.EPI-14-0207 PMC4257885 25192704

[22] Stokland T Liu J-F Ironside JW Ellison DW Taylor R Robinson KJ. A Multivariate Analysis of Factors Determining Tumor Progression in Childhood Low-Grade Glioma: A Population-Based Cohort Study (CCLG CNS9702). Neuro-Oncol (2010) 12(12):1257–68. 10.1093/neuonc/noq092 PMC3018938 20861086

[23] Mistry M Zhukova N Merico D Rakopoulos P Krishnatry R Shago M. BRAF Mutation and CDKN2A Deletion Define a Clinically Distinct Subgroup of Childhood Secondary High-Grade Glioma. J Clin Oncol Off J Am Soc Clin Oncol (2015) 33(9):1015–22. 10.1200/JCO.2014.58.3922 PMC4356711 25667294

[24] Cacciotti C Fleming A Ramaswamy V. Advances in the Molecular Classification of Pediatric Brain Tumors: A Guide to the Galaxy. J Pathol (2020) 251(3):249–61. 10.1002/path.5457 32391583

[25] Sturm D Pfister SM Jones DTW. Pediatric Gliomas: Current Concepts on Diagnosis, Biology, and Clinical Management. J Clin Oncol Off J Am Soc Clin Oncol (2017) 35(21):2370–7. 10.1200/JCO.2017.73.0242 28640698

[26] Lassaletta A Zapotocky M Mistry M Ramaswamy V Honnorat M Krishnatry R. Therapeutic and Prognostic Implications of BRAF V600E in Pediatric Low-Grade Gliomas. J Clin Oncol (2017) 35(25):2934–41. 10.1200/JCO.2016.71.8726 PMC5791837 28727518

[27] French JA Lawson JA Yapici Z Ikeda H Polster T Nabbout R. Adjunctive Everolimus Therapy for Treatment-Resistant Focal-Onset Seizures Associated With Tuberous Sclerosis (EXIST-3): A Phase 3, Randomised, Double-Blind, Placebo-Controlled Study. Lancet Lond Engl (2016) 388(10056):2153–63. 10.1016/S0140-6736(16)31419-2 27613521

[28] Bax DA Mackay A Little SE Carvalho D Viana–Pereira M Tamber N. A Distinct Spectrum Of Copy Number Aberrations In Paediatric High Grade Gliomas. Clin Cancer Res Off J Am Assoc Cancer Res (2010) 16(13):3368–77. 10.1158/1078-0432.CCR-10-0438 PMC2896553 20570930

[29] Zarghooni M Bartels U Lee E Buczkowicz P Morrison A Huang A. Whole-Genome Profiling of Pediatric Diffuse Intrinsic Pontine Gliomas Highlights Platelet-Derived Growth Factor Receptor α and Poly (ADP-Ribose) Polymerase as Potential Therapeutic Targets. J Clin Oncol (2010) 28(8):1337–44. 10.1200/JCO.2009.25.5463 20142589

[30] Schwartzentruber J Korshunov A Liu X-Y Jones DTW Pfaff E Jacob K. Driver Mutations in Histone H3.3 and Chromatin Remodelling Genes in Paediatric Glioblastoma. Nature (2012) 482(7384):226–31. 10.1038/nature10833 22286061

[31] Wu G Broniscer A McEachron TA Lu C Paugh BS Becksfort J. Somatic Histone H3 Alterations in Pediatric Diffuse Intrinsic Pontine Gliomas and Non-Brainstem Glioblastomas. Nat Genet (2012) 44(3):251–3. 10.1038/ng.1102 PMC3288377 22286216

[32] Korshunov A Capper D Reuss D Schrimpf D Ryzhova M Hovestadt V. Histologically Distinct Neuroepithelial Tumors With Histone 3 G34 Mutation Are Molecularly Similar and Comprise a Single Nosologic Entity. Acta Neuropathol (Berl.) (2016) 131(1):137–46. 10.1007/s00401-015-1493-1 26482474

[33] Doebele RC Drilon A Paz–Ares L Siena S Shaw AT Farago AF. Entrectinib in Patients With Advanced or Metastatic NTRK Fusion-Positive Solid Tumours: Integrated Analysis of Three Phase 1-2 Trials. Lancet Oncol (2020) 21(2):271–82. 10.1016/S1470-2045(19)30691-6 PMC7461630 31838007

[34] Gottardo NG Gajjar A. Chemotherapy for Malignant Brain Tumors of Childhood. J Child Neurol (2008) 23(10):1149–59. 10.1177/0883073808321765 PMC2695990 18952581

[35] Khuong-Quang D-A Buczkowicz P Rakopoulos P Liu X-Y Fontebasso AM Bouffet E. K27M Mutation in Histone H3.3 Defines Clinically and Biologically Distinct Subgroups of Pediatric Diffuse Intrinsic Pontine Gliomas. Acta Neuropathol (Berl.) (2012) 124(3):439–47. 10.1007/s00401-012-0998-0 PMC3422615 22661320

[36] Pollack IF Agnihotri S Broniscer A. Childhood Brain Tumors: Current Management, Biological Insights, and Future Directions. J Neurosurg Pediatr (2019) 23(3):261–73. 10.3171/2018.10.PEDS18377 PMC6823600 30835699

[37] Millard NE De Braganca KC. Medulloblastoma. J Child Neurol (2016) vol:1341–53. 10.1177/0883073815600866 PMC4995146 26336203

[38] Louis DN Perry A Reifenberger G von Deimling A Figarella–Branger D Cavenee WK. The 2016 World Health Organization Classification of Tumors of the Central Nervous System: A Summary. Acta Neuropathol (Berl.) (2016) 131(6):803–20. 10.1007/s00401-016-1545-1 27157931

[39] Ramaswamy V Remke M Bouffet E Bailey S Clifford SC Doz F. Risk Stratification of Childhood Medulloblastoma in the Molecular Era: The Current Consensus. Acta Neuropathol (Berl.) (2016) 131(6):821–31. 10.1007/s00401-016-1569-6 PMC4867119 27040285

[40] Remke M Ramaswamy V. Infant Medulloblastoma - Learning New Lessons From Old Strata. Nat Rev Clin Oncol (2018) 15(11):659–60. 10.1038/s41571-018-0071-6 30030473

[41] Mulhern RK Palmer SL Merchant TE Wallace D Kocak M Brouwers P. Neurocognitive Consequences of Risk-Adapted Therapy for Childhood Medulloblastoma. J Clin Oncol Off J Am Soc Clin Oncol (2005) 23(24):5511–9. 10.1200/JCO.2005.00.703 16110011

[42] Mack SC Taylor MD. Put Away Your Microscopes: The Ependymoma Molecular Era has Begun. Curr Opin Oncol (2017) 29(6):443–7. 10.1097/CCO.0000000000000411 PMC6003664 28885433

[43] Parker M Mohankumar KM Punchihewa C Weinlich R Dalton JD Li Y. C11orf95-RELA Fusions Drive Oncogenic NF-κb Signaling in Ependymoma. Nature (2014) 506(7489):451–5. 10.1038/nature13109 PMC4050669 24553141

[44] Pajtler KW Witt H Sill M Jones DTW Hovestadt V Kratochwil F. Molecular Classification of Ependymal Tumors Across All Cns Compartments, Histopathological Grades, and Age Groups. Cancer Cell (2015) 27(5):728–43. 10.1016/j.ccell.2015.04.002 PMC4712639 25965575

[45] Zapotocky M Beera K Adamski J Laperierre N Guger S Janzen L. Survival and Functional Outcomes of Molecularly Defined Childhood Posterior Fossa Ependymoma: Cure at a Cost. Cancer (2019) 125(11):1867–76. 10.1002/cncr.31995 PMC6508980 30768777

[46] Mostoufi-Moab S Grimberg A. Pediatric Brain Tumor Treatment: Growth Consequences and Their Management. Pediatr Endocrinol Rev PER (2010) 8(1):6–17. 21037539 PMC4148717

[47] Sengupta S Pomeranz Krummel D Pomeroy S. The Evolution of Medulloblastoma Therapy to Personalized Medicine. F1000Research (2017) 6:490. 10.12688/f1000research.10859.1 28713553 PMC5490254

[48] Jussila M-P Remes T Anttonen J Harila–Saari A Niinimäki J Pokka T. Late Vertebral Side Effects in Long-Term Survivors of Irradiated Childhood Brain Tumor. PloS One (2018) 13(12):e0209193. 10.1371/journal.pone.0209193 30562369 PMC6298650

[49] Kolodkin AL Levengood DV Rowe EG Tai YT Giger RJ Ginty DD. Neuropilin Is a Semaphorin III Receptor. Cell (1997) 90(4):753–62. 10.1016/s0092-8674(00)80535-8 9288754

[50] Koch S Claesson-Welsh L. Signal Transduction by Vascular Endothelial Growth Factor Receptors. Cold Spring Harb Perspect Med (2012) 2(7):a006502. 10.1101/cshperspect.a006502 22762016 PMC3385940

[51] Gaur P Bielenberg DR Samuel S Bose D Zhou Y Gray MJ. Role of Class 3 Semaphorins and Their Receptors in Tumor Growth and Angiogenesis. Clin Cancer Res Off J Am Assoc Cancer Res (2009) 15(22):6763–70. 10.1158/1078-0432.CCR-09-1810 19887479

[52] Pellet-Many C Frankel P Jia H Zachary I. Neuropilins: Structure, Function and Role in Disease. Biochem J (2008) 411(2):211–26. 10.1042/BJ20071639 18363553

[53] Wild JRL Staton CA Chapple K Corfe BM. Neuropilins: Expression and Roles in the Epithelium. Int J Exp Pathol (2012) 93(2):81–103. 10.1111/j.1365-2613.2012.00810.x 22414290 PMC3385701

[54] Prahst C Héroult M Lanahan AA Uziel N Kessler O Shraga–Heled N. Neuropilin-1-Vegfr-2 Complexing Requires the PDZ-Binding Domain of Neuropilin-1. J Biol Chem (2008) 283(37):25110–4. 10.1074/jbc.C800137200 PMC2533068 18628209

[55] Grun D Adhikary G Eckert RL. VEGF-a Acts Via Neuropilin-1 to Enhance Epidermal Cancer Stem Cell Survival and Formation of Aggressive and Highly Vascularized Tumors. Oncogene (2016) 35(33):4379–87. 10.1038/onc.2015.507 PMC4959993 26804163

[56] Valdembri D Caswell PT Anderson KI Schwarz JP König I Astanina E. Neuropilin-1/Gipc1 Signaling Regulates α5β1 Integrin Traffic and Function in Endothelial Cells. PloS Biol (2009) 7(1):e25. 10.1371/journal.pbio.1000025 19175293 PMC2631072

[57] Chauvet S Cohen S Yoshida Y Fekrane L Livet J Gayet O. Gating of Sema3E/PlexinD1 Signaling by Neuropilin-1 Switches Axonal Repulsion to Attraction During Brain Development. Neuron (2007) 56(5):807–22. 10.1016/j.neuron.2007.10.019 PMC2700040 18054858

[58] Pan Q Chathery Y Wu Y Rathore N Tong RK Peale F. Neuropilin-1 Binds to VEGF121 and Regulates Endothelial Cell Migration and Sprouting. J Biol Chem (2007) 282(33):24049–56. 10.1074/jbc.M703554200 17575273

[59] Gu C Limberg BJ Whitaker GB Perman B Leahy DJ Rosenbaum JS. Characterization of Neuropilin-1 Structural Features That Confer Binding to Semaphorin 3A and Vascular Endothelial Growth Factor 165. J Biol Chem (2002) 277(20):18069–76. 10.1074/jbc.M201681200 11886873

[60] West DC Rees CG Duchesne L Patey SJ Terry CJ Turnbull JE. Interactions of Multiple Heparin Binding Growth Factors With Neuropilin-1 and Potentiation of the Activity of Fibroblast Growth Factor-2. J Biol Chem (2005) 280(14):13457–64. 10.1074/jbc.M410924200 15695515

[61] Hu B Guo P Bar–Joseph I Imanishi Y Jarzynka M Bogler O. Neuropilin-1 Promotes Human Glioma Progression Through Potentiating the Activity of the HGF/SF Autocrine Pathway. Oncogene (2007) 26(38):5577–86. 10.1038/sj.onc.1210348 PMC2846324 17369861

[62] Matsushita A Götze T Korc M. Hepatocyte Growth Factor-Mediated Cell Invasion in Pancreatic Cancer Cells Is Dependent on Neuropilin-1. Cancer Res (2007) 67(21):10309–16. 10.1158/0008-5472.CAN-07-3256 17974973

[63] Banerjee S Sengupta K Dhar K Mehta S D’Amore PA Dhar G. Breast Cancer Cells Secreted Platelet-Derived Growth Factor-Induced Motility of Vascular Smooth Muscle Cells Is Mediated Through Neuropilin-1. Mol Carcinog (2006) 45(11):871–80. 10.1002/mc.20248 16847823

[64] Kofler N Simons M. The Expanding Role of Neuropilin: Regulation of Vascular Tgfβ and PDGF Signaling. Curr Opin Hematol (2016) 23(3):260–7. 10.1097/MOH.0000000000000233 PMC4957701 26849476

[65] Glinka Y Prud’homme GJ. Neuropilin-1 Is a Receptor for Transforming Growth Factor β-1, Activates Its Latent Form, and Promotes Regulatory T Cell Activity. J Leukoc Biol (2008) 84(1):302–10. 10.1189/jlb.0208090 PMC2504713 18436584

[66] Kofler N Simons M. The Expanding Role of Neuropilin: Regulation of Transforming Growth Factor-β and Platelet-Derived Growth Factor Signaling in the Vasculature. Curr Opin Hematol (2016) 23(3):260–7. 10.1097/MOH.0000000000000233 PMC4957701 26849476

[67] Ball SG Bayley C Shuttleworth CA Kielty CM. Neuropilin-1 Regulates Platelet-Derived Growth Factor Receptor Signalling in Mesenchymal Stem Cells. Biochem J (2010) 427(Pt 1):29–40. 10.1042/BJ20091512 20102335 PMC3441150

[68] Glinka Y Stoilova S Mohammed N Prud’homme GJ. Neuropilin-1 Exerts Co-Receptor Function for TGF-Beta-1 on the Membrane of Cancer Cells and Enhances Responses to Both Latent and Active TGF-Beta. Carcinogenesis (2011) 32(4):613–21. 10.1093/carcin/bgq281 21186301

[69] Hirota S Clements TP Tang LK Morales JE Lee HS Oh SP. Neuropilin 1 Balances β8 Integrin-Activated Tgfβ Signaling to Control Sprouting Angiogenesis in the Brain. Dev Camb Engl (2015) 142(24):4363–73. 10.1242/dev.113746 PMC4689212 26586223

[70] Kawakami T Tokunaga T Hatanaka H Kijima H Yamazaki H Abe Y. Neuropilin 1 and Neuropilin 2 Co-Expression Is Significantly Correlated With Increased Vascularity and Poor Prognosis In Nonsmall Cell Lung Carcinoma. Cancer (2002) 95(10):2196–201. 10.1002/cncr.10936 12412174

[71] Chu W Song X Yang X Ma L Zhu J He M. Neuropilin-1 Promotes Epithelial-to-Mesenchymal Transition by Stimulating Nuclear Factor-Kappa B and Is Associated With Poor Prognosis in Human Oral Squamous Cell Carcinoma. PloS One (2014) 9(7):e101931. 10.1371/journal.pone.0101931 24999732 PMC4084996

[72] Hamerlik P Lathia JD Rasmussen R Wu Q Bartkova J Lee M. Autocrine VEGF-VEGFR2-Neuropilin-1 Signaling Promotes Glioma Stem-Like Cell Viability and Tumor Growth. J Exp Med (2012) 209(3):507–20. 10.1084/jem.20111424 PMC3302235 22393126

[73] Baumgarten P Blank A-E Franz K Hattingen E Dunst M Zeiner P. Differential Expression of Vascular Endothelial Growth Factor A, Its Receptors VEGFR-1, -2, and -3 and Co-Receptors Neuropilin-1 and -2 Does Not Predict Bevacizumab Response in Human Astrocytomas. Neuro-Oncol (2016) 18(2):173–83. 10.1093/neuonc/nov288 PMC4724185 26627848

[74] Clemessy M Janzer RC Lhermitte B Gasc J-M Juillerat-Jeanneret L. Expression of Dual Angiogenic/Neurogenic Growth Factors in Human Primary Brain Tumors. J Neurooncol (2012) 107(1):29–36. 10.1007/s11060-011-0715-1 21979892

[75] Zhao H Hou C Hou A Zhu D. Concurrent Expression of VEGF-C and Neuropilin-2 Is Correlated With Poor Prognosis in Glioblastoma. Tohoku J Exp Med (2016) 238(2):85–91. 10.1620/tjem.238.85 26753562

[76] Zhang G Chen L Sun K Khan AA Yan J Liu H. Neuropilin-1 (Nrp-1)/GIPC1 Pathway Mediates Glioma Progression. Tumour Biol J Int Soc Oncodevelopmental Biol Med (2016) 37(10):13777–88. 10.1007/s13277-016-5138-3 27481513

[77] Zhang G Chen L Khan AA Li B Gu B Lin F. miRNA-124-3p/Neuropilin-1(NRP-1) Axis Plays an Important Role in Mediating Glioblastoma Growth and Angiogenesis. Int J Cancer (2018) 143(3):635–44. 10.1002/ijc.31329 29457830

[78] Jacob L Sawma P Garnier N Meyer LAT Fritz J Hussenet T. Inhibition of PlexA1-mediated Brain Tumor Growth and Tumor-Associated Angiogenesis Using a Transmembrane Domain Targeting Peptide. Oncotarget (2016) 7(36):57851–65. 10.18632/oncotarget.11072 PMC5295395 27506939

[79] Lee J Kim E Ryu S-W Choi C Choi K. Combined Inhibition of Vascular Endothelial Growth Factor Receptor Signaling With Temozolomide Enhances Cytotoxicity Against Human Glioblastoma Cells Via Downregulation of Neuropilin-1. J Neurooncol (2016) 128(1):29–34. 10.1007/s11060-016-2091-3 26951556

[80] Angom RS Mondal SK Wang F Madamsetty VS Wang E Dutta SK. Ablation of Neuropilin-1 Improves the Therapeutic Response in Conventional Drug-Resistant Glioblastoma Multiforme. Oncogene (2020) 39(48):7114–26. 10.1038/s41388-020-01462-1 33005016

[81] Kwiatkowski SC Guerrero PA Hirota S Chen Z Morales JE Aghi M. Neuropilin-1 Modulates Tgfβ Signaling to Drive Glioblastoma Growth and Recurrence After Anti-Angiogenic Therapy. PloS One (2017) 12(9):e0185065. 10.1371/journal.pone.0185065 28938007 PMC5609745

[82] Gong C Valduga J Chateau A Richard M Pellegrini–Moïse N Barberi–Heyob M. Stimulation of Medulloblastoma Stem Cells Differentiation by a Peptidomimetic Targeting Neuropilin-1. Oncotarget (2018) 9(20):15312–25. 10.18632/oncotarget.24521 PMC5880606 29632646

[83] Yogi K Sridhar E Goel N Jalali R Goel A Moiyadi A. MiR-148a, a microRNA Upregulated in the WNT Subgroup Tumors, Inhibits Invasion and Tumorigenic Potential of Medulloblastoma Cells by Targeting Neuropilin 1. Oncoscience (2015) 2(4):334–48. 10.18632/oncoscience.137 PMC4468320 26097868

[84] Ge X Milenkovic L Suyama K Hartl T Purzner T Winans A. Phosphodiesterase 4D Acts Downstream of Neuropilin to Control Hedgehog Signal Transduction and the Growth of Medulloblastoma. eLife (2015) 4:e07068. 10.7554/eLife.07068 PMC4569902 26371509

[85] Hayden Gephart MG Sophie Su Y Bandara S Tsai F-C Hong J Conley N. Neuropilin-2 Contributes to Tumorigenicity in a Mouse Model of Hedgehog Pathway Medulloblastoma. J Neurooncol (2013) 115(2):161–8. 10.1007/s11060-013-1216-1 PMC6783276 24026530

[86] Ishizuka Y Koshinaga T Hirano T Nagasaki–Maeoka E Watanabe Y Hoshi R. NRP1 Knockdown Promotes the Migration and Invasion of Human Neuroblastoma-Derived SK−N−AS Cells *Via* the Activation of β1 Integrin Expression. Int J Oncol (2018) 53(1):159–66. 10.3892/ijo.2018.4397 29750423

[87] Clarke MF Dick JE Dirks PB Eaves CJ Jamieson CHM Jones DL. Cancer Stem Cells–Perspectives on Current Status and Future Directions: AACR Workshop on Cancer Stem Cells. Cancer Res (2006) 66(19):9339–44. 10.1158/0008-5472.CAN-06-3126 16990346

[88] Mercurio AM. Vegf/Neuropilin Signaling in Cancer Stem Cells. Int J Mol Sci (2019) 20(3):490. 10.3390/ijms20030490 PMC6387347 30678134

[89] Nunes T Hamdan D Leboeuf C El Bouchtaoui M Gapihan G Nguyen TT. Targeting Cancer Stem Cells to Overcome Chemoresistance. Int J Mol Sci (2018) 19(12):4036. 10.3390/ijms19124036 PMC6321478 30551640

[90] Cojoc M Mäbert K Muders MH Dubrovska A. A Role for Cancer Stem Cells in Therapy Resistance: Cellular and Molecular Mechanisms. Semin Cancer Biol (2015) 31:16–27. 10.1016/j.semcancer.2014.06.004 24956577

[91] Garcia-Mayea Y Mir C Masson F Paciucci R LLeonart ME. Insights Into New Mechanisms and Models of Cancer Stem Cell Multidrug Resistance. Semin Cancer Biol (2020) 60:166–80. 10.1016/j.semcancer.2019.07.022 31369817

[92] Yang L Shi P Zhao G Xu J Peng W Zhang J. Targeting Cancer Stem Cell Pathways for Cancer Therapy. Signal Transduct Target Ther (2020) 5(1):8. 10.1038/s41392-020-0110-5 32296030 PMC7005297

[93] Mani SA Guo W Liao M-J Eaton ENG Ayyanan A Zhou AY. The Epithelial-Mesenchymal Transition Generates Cells With Properties of Stem Cells. Cell (2008) 133(4):704–15. 10.1016/j.cell.2008.03.027 PMC2728032 18485877

[94] Al-Hajj M Wicha MS Benito-Hernandez A Morrison SJ Clarke MF. Prospective Identification of Tumorigenic Breast Cancer Cells. Proc Natl Acad Sci USA (2003) 100(7):3983–8. 10.1073/pnas.0530291100 PMC153034 12629218

[95] Ricci-Vitiani L Lombardi DG Pilozzi E Biffoni M Todaro M Peschle C. Identification and Expansion of Human Colon-Cancer-Initiating Cells. Nature (2007) 445(7123):111–5. 10.1038/nature05384 17122771

[96] Ignatova TN Kukekov VG Laywell ED Suslov ON Vrionis FD Steindler DA. Human Cortical Glial Tumors Contain Neural Stem-Like Cells Expressing Astroglial and Neuronal Markers *In Vitro* . Glia (2002) 39(3):193–206. 10.1002/glia.10094 12203386

[97] Bapat SA Mali AM Koppikar CB Kurrey NK. Stem and Progenitor-Like Cells Contribute to the Aggressive Behavior of Human Epithelial Ovarian Cancer. Cancer Res (2005) 65(8):3025–9. 10.1158/0008-5472.CAN-04-3931 15833827

[98] Singh SK Hawkins C Clarke ID Squire JA Bayani J Hide T. Identification of Human Brain Tumour Initiating Cells. Nature (2004) 432(7015):396–401. 10.1038/nature03128 15549107

[99] Hemmati HD Nakano I Lazareff JA Masterman–Smith M Geschwind DH Bronner–Fraser M. Cancerous Stem Cells can Arise From Pediatric Brain Tumors. Proc Natl Acad Sci USA (2003) 100(25):15178–83. 10.1073/pnas.2036535100 PMC299944 14645703

[100] Singh SK Clarke ID Hide T Dirks PB. Cancer Stem Cells in Nervous System Tumors. Oncogene (2004) 23(43):7267–73. 10.1038/sj.onc.1207946 15378086

[101] Zhang L He X Liu X Zhang F Huang LF Potter AS. Single-Cell Transcriptomics in Medulloblastoma Reveals Tumor-Initiating Progenitors and Oncogenic Cascades During Tumorigenesis and Relapse. Cancer Cell (2019) 36(3):302–18.e7. 10.1016/j.ccell.2019.07.009 31474569 PMC6760242

[102] Read T-A Fogarty MP Markant SL McLendon RE Wei Z Ellison DW. Identification of CD15 as a Marker for Tumor-Propagating Cells in a Mouse Model of Medulloblastoma. Cancer Cell (2009) 15(2):135–47. 10.1016/j.ccr.2008.12.016 PMC2664097 19185848

[103] Hambardzumyan D Becher OJ Rosenblum MK Pandolfi PP Manova-Todorova K Holland EC. PI3K Pathway Regulates Survival of Cancer Stem Cells Residing in the Perivascular Niche Following Radiation in Medulloblastoma *In Vivo* . Genes Dev (2008) 22(4):436–48. 10.1101/gad.1627008 PMC2238666 18281460

[104] Vanner RJ Remke M Gallo M Selvadurai HJ Coutinho F Lee L. Quiescent sox2(+) Cells Drive Hierarchical Growth and Relapse in Sonic Hedgehog Subgroup Medulloblastoma. Cancer Cell (2014) 26(1):33–47. 10.1016/j.ccr.2014.05.005 24954133 PMC4441014

[105] Friedman GK Raborn J Kelly VM Cassady KA Markert JM Gillespie GY. Pediatric Glioma Stem Cells: Biologic Strategies for Oncolytic HSV Virotherapy. Front Oncol (2013) 3:28. 10.3389/fonc.2013.00028 23450706 PMC3584319

[106] Manoranjan B Venugopal C McFarlane N Doble BW Dunn SE Scheinemann K. Medulloblastoma Stem Cells: Modeling Tumor Heterogeneity. Cancer Lett (2013) 338(1):23–31. 10.1016/j.canlet.2012.07.010 22796365

[107] Liu W Wu T Dong X Zeng YA. Neuropilin-1 Is Upregulated by Wnt/β-Catenin Signaling and Is Important for Mammary Stem Cells. Sci Rep (2017) 7(1):10941. 10.1038/s41598-017-11287-w 28887477 PMC5591238

[108] Zhang L Wang H Li C Zhao Y Wu L Du X. Vegf-A/Neuropilin 1 Pathway Confers Cancer Stemness *Via* Activating Wnt/β-Catenin Axis in Breast Cancer Cells. Cell Physiol Biochem Int J Exp Cell Physiol Biochem Pharmacol (2017) 44(3):1251–62. 10.1159/000485455 29179185

[109] Grun D Adhikary G Eckert RL. NRP-1 Interacts With GIPC1 and α6/β4-Integrins to Increase YAP1/Δnp63α-Dependent Epidermal Cancer Stem Cell Survival. Oncogene (2018) 37(34):4711–22. 10.1038/s41388-018-0290-4 PMC6381998 29755126

[110] Elaimy AL Guru S Chang C Ou J Amante JJ Zhu LJ. VEGF-Neuropilin-2 Signaling Promotes Stem-Like Traits in Breast Cancer Cells by TAZ-mediated Repression of the Rac Gap β2-Chimaerin. Sci Signal (2018) 11(528):eaao6897. 10.1126/scisignal.aao6897 29717062 PMC6592619

[111] Goel HL Pursell B Chang C Shaw LM Mao J Simin K. GLI1 Regulates a Novel Neuropilin-2/α6β1 Integrin Based Autocrine Pathway That Contributes to Breast Cancer Initiation. EMBO Mol Med (2013) 5(4):488–508. 10.1002/emmm.201202078 23436775 PMC3628099

[112] Thirant C Bessette B Varlet P Puget S Cadusseau J Tavares SDR. Clinical Relevance of Tumor Cells With Stem-Like Properties in Pediatric Brain Tumors. PloS One (2011) 6(1):e16375. 10.1371/journal.pone.0016375 21297991 PMC3030582

[113] Man J Shoemake J Zhou W Fang X Wu Q Rizzo A. Sema3c Promotes the Survival and Tumorigenicity of Glioma Stem Cells Through Rac1 Activation. Cell Rep (2014) 9(5):1812–26. 10.1016/j.celrep.2014.10.055 PMC4268066 25464848

[114] Mitra A Mishra L Li S. Emt, CTCs and CSCs in Tumor Relapse and Drug-Resistance. Oncotarget (2015) 6(13):10697–711. 10.18632/oncotarget.4037 PMC4484413 25986923

[115] Garg N Bakhshinyan D Venugopal C Mahendram S Rosa DA Vijayakumar T. CD133+ Brain Tumor-Initiating Cells Are Dependent on STAT3 Signaling to Drive Medulloblastoma Recurrence. Oncogene (2017) 36(5):606–17. 10.1038/onc.2016.235 PMC5541269 27775079

[116] Bruder D Probst‐Kepper M Westendorf AM Geffers R Beissert S Loser K. Frontline: Neuropilin-1: A Surface Marker of Regulatory T Cells. Eur J Immunol (2004) 34(3):623–30. 10.1002/eji.200324799 14991591

[117] Catalano A. The Neuroimmune Semaphorin-3a Reduces Inflammation and Progression of Experimental Autoimmune Arthritis. J Immunol (2010) 185(10):6373–83. 10.4049/jimmunol.0903527 20937848

[118] Curreli S Wong BS Latinovic O Konstantopoulos K Stamatos NM. Class 3 Semaphorins Induce F-Actin Reorganization in Human Dendritic Cells: Role in Cell Migration. J Leukoc Biol (2016) 100(6):1323–34. 10.1189/jlb.2A1114-534R PMC5109998 27406993

[119] Ji J-D Park-Min K-H Ivashkiv LB. Expression and Function of Semaphorin 3A and Its Receptors in Human Monocyte-Derived Macrophages. Hum Immunol (2009) 70(4):211–7. 10.1016/j.humimm.2009.01.026 PMC4811352 19480842

[120] Nakayama H Bruneau S Kochupurakkal N Coma S Briscoe DM Klagsbrun M. Regulation of Mtor Signaling by Semaphorin 3f-Neuropilin 2 Interactions *in Vitro* and *In Vivo* . Sci Rep (2015) 5:11789. 10.1038/srep11789 26156437 PMC4496725

[121] Stepanova OI Krylov AV Lioudyno VI Kisseleva EP. Gene Expression for VEGF-A, Vegf-C, and Their Receptors in Murine Lymphocytes and Macrophages. Biochem Biokhimiia (2007) 72(11):1194–8. 10.1134/s0006297907110041 18205601

[122] Takamatsu H Takegahara N Nakagawa Y Tomura M Taniguchi M Friedel RH. Semaphorins Guide the Entry of Dendritic Cells Into the Lymphatics by Activating Myosin II. Nat Immunol (2010) 11(7):594–600. 10.1038/ni.1885 20512151 PMC3045806

[123] Tordjman R Lepelletier Y Lemarchandel V Cambot M Gaulard P Hermine O. A Neuronal Receptor, Neuropilin-1, Is Essential for the Initiation of the Primary Immune Response. Nat Immunol (2002) 3(5):477–82. 10.1038/ni789 11953749

[124] Sarris M Andersen KG Randow F Mayr L Betz AG. Neuropilin-1 Expression on Regulatory T Cells Enhances Their Interactions With Dendritic Cells During Antigen Recognition. Immunity (2008) 28(3):402–13. 10.1016/j.immuni.2008.01.012 PMC2726439 18328743

[125] Bourbié-Vaudaine S Blanchard N Hivroz C Roméo P-H. Dendritic Cells Can Turn Cd4+ T Lymphocytes Into Vascular Endothelial Growth Factor-Carrying Cells by Intercellular Neuropilin-1 Transfer. J Immunol (2006) 177(3):1460–9. 10.4049/jimmunol.177.3.1460 16849452

[126] Aung NY Ohe R Meng H Kabasawa T Yang S Kato T. Specific Neuropilins Expression in Alveolar Macrophages Among Tissue-Specific Macrophages. PloS One (2016) 11(2):e0147358. 10.1371/journal.pone.0147358 26900851 PMC4764655

[127] Casazza A Laoui D Wenes M Rizzolio S Bassani N Mambretti M. Impeding Macrophage Entry Into Hypoxic Tumor Areas by Sema3A/Nrp1 Signaling Blockade Inhibits Angiogenesis and Restores Antitumor Immunity. Cancer Cell (2013) 24(6):695–709. 10.1016/j.ccr.2013.11.007 24332039

[128] Roy S Bag AK Dutta S Polavaram NS Islam R Schellenburg S. Macrophage-Derived Neuropilin-2 Exhibits Novel Tumor-Promoting Functions. Cancer Res (2018) 78(19):5600–17. 10.1158/0008-5472.CAN-18-0562 PMC6168405 30111533

[129] Miyauchi JT Chen D Choi M Nissen JC Shroyer KR Djordevic S. Ablation of Neuropilin 1 From Glioma-Associated Microglia and Macrophages Slows Tumor Progression. Oncotarget (2016) 7(9):9801–14. 10.18632/oncotarget.6877 PMC4891085 26755653

[130] Miyauchi JT Caponegro MD Chen D Choi MK Li M Tsirka SE. Deletion of Neuropilin 1 From Microglia or Bone Marrow-Derived Macrophages Slows Glioma Progression. Cancer Res (2018) 78(3):685–94. 10.1158/0008-5472.CAN-17-1435 PMC5887044 29097606

[131] Lepelletier Y Smaniotto S Hadj–Slimane R Villa–Verde DMS Nogueira AC Dardenne M. Control of Human Thymocyte Migration by Neuropilin-1/Semaphorin-3A-Mediated Interactions. Proc Natl Acad Sci USA (2007) 104(13):5545–50. 10.1073/pnas.0700705104 PMC1838472 17369353

[132] Mendes-da-Cruz DA Brignier AC Asnafi V Baleydier F Messias CV Lepelletier Y. Semaphorin 3F and Neuropilin-2 Control the Migration of Human T-Cell Precursors. PloS One (2014) 9(7):e103405. 10.1371/journal.pone.0103405 25068647 PMC4113369

[133] Solomon BD Mueller C Chae W-J Alabanza LM Bynoe MS. Neuropilin-1 Attenuates Autoreactivity in Experimental Autoimmune Encephalomyelitis. Proc Natl Acad Sci USA (2011) 108(5):2040–5. 10.1073/pnas.1008721108 PMC3033275 21245328

[134] Kerros C Tripathi SC Zha D Mehrens JM Sergeeva A Philips AV. Neuropilin-1 Mediates Neutrophil Elastase Uptake and Cross-Presentation in Breast Cancer Cells. J Biol Chem (2017) 292(24):10295–305. 10.1074/jbc.M116.773051 PMC5473232 28468826

[135] Leclerc M Voilin E Gros G Corgnac S de Montpréville V Validire P. Regulation of Antitumour CD8 T-Cell Immunity and Checkpoint Blockade Immunotherapy by Neuropilin-1. Nat Commun (2019) 10(1):3345. 10.1038/s41467-019-11280-z 31350404 PMC6659631

[136] Delgoffe GM Woo S-R Turnis ME Gravano DM Guy C Overacre AE. Regulatory T Cell Stability Is Maintained by a Neuropilin-1:semaphorin-4a Axis. Nature (2013) 501(7466):252–6. 10.1038/nature12428 PMC3867145 23913274

[137] Podojil JR Chiang M-Y Ifergan I Copeland R Liu LN Maloveste S. B7-H4 Modulates Regulatory Cd4+ T Cell Induction and Function Via Ligation of a Semaphorin 3a/Plexin A4/Neuropilin-1 Complex. J Immunol Baltim Md 1950 (2018) 201(3):897–907. 10.4049/jimmunol.1700811 PMC6894186 29898965

[138] Hansen W Hutzler M Abel S Alter C Stockmann C Kliche S. Neuropilin 1 Deficiency on CD4+Foxp3+ Regulatory T Cells Impairs Mouse Melanoma Growth. J Exp Med (2012) 209(11):2001–16. 10.1084/jem.20111497 PMC3478934 23045606

[139] Overacre-Delgoffe AE Chikina M Dadey RE Yano H Brunazzi EA Shayan G. Interferon-γ Drives Treg Fragility to Promote Anti-Tumor Immunity. Cell (2017) 169(6):1130–41.e11. 10.1016/j.cell.2017.05.005 28552348 PMC5509332

[140] Powell J Mota F Steadman D Soudy C Miyauchi JT Crosby S. Small Molecule Neuropilin-1 Antagonists Combine Antiangiogenic and Antitumor Activity With Immune Modulation Through Reduction of Transforming Growth Factor Beta (Tgfβ) Production in Regulatory T-Cells. J Med Chem (2018) 61(9):4135–54. 10.1021/acs.jmedchem.8b00210 PMC5957473 29648813

[141] Chaudhary B Elkord E. Novel Expression of Neuropilin 1 on Human Tumor-Infiltrating Lymphocytes in Colorectal Cancer Liver Metastases. Expert Opin Ther Targets (2015) 19(2):147–61. 10.1517/14728222.2014.977784 25351619

[142] Candeias SM Gaipl US. The Immune System in Cancer Prevention, Development and Therapy. Anticancer Agents Med Chem (2016) 16(1):101–7. 10.2174/1871520615666150824153523 26299661

[143] Muenst S Läubli H Soysal SD Zippelius A Tzankov A Hoeller S. The Immune System and Cancer Evasion Strategies: Therapeutic Concepts. J Intern Med (2016) 279(6):541–62. 10.1111/joim.12470 26748421

[144] Bockmayr M Mohme M Klauschen F Winkler B Budczies J Rutkowski S. Subgroup-Specific Immune and Stromal Microenvironment in Medulloblastoma. Oncoimmunology (2018) 7(9):e1462430. 10.1080/2162402X.2018.1462430 30228931 PMC6140816

[145] Weekes CD Beeram M Tolcher AW Papadopoulos KP Gore L Hegde P. A Phase I Study of the Human Monoclonal Anti-NRP1 Antibody MNRP1685A in Patients With Advanced Solid Tumors. Invest New Drugs (2014) 32(4):653–60. 10.1007/s10637-014-0071-z 24604265

[146] Lassen U Nielsen DL Sørensen M Winstedt L Niskanen T Stenberg Y. A Phase I, Dose-Escalation Study of TB-403, a Monoclonal Antibody Directed Against PlGF, in Patients With Advanced Solid Tumours. Br J Cancer (2012) 106(4):678–84. 10.1038/bjc.2011.609 PMC3322959 22333707

